# Drought Sensitivity of Norway Spruce at the Species’ Warmest Fringe: Quantitative and Molecular Analysis Reveals High Genetic Variation Among and Within Provenances

**DOI:** 10.1534/g3.117.300524

**Published:** 2018-02-09

**Authors:** Carlos Trujillo-Moya, Jan-Peter George, Silvia Fluch, Thomas Geburek, Michael Grabner, Sandra Karanitsch-Ackerl, Heino Konrad, Konrad Mayer, Eva Maria Sehr, Elisabeth Wischnitzki, Silvio Schueler

**Affiliations:** * Department of Forest Genetics and; § Department of Forest Growth and Silviculture, Federal Research and Training Centre for Forests, Natural Hazards and Landscapes, 1131 Vienna, Austria; † Center for Health & Bioresources, AIT Austrian Institute of Technology GmbH, 3430 Tulln, Austria, and; ‡ Institute of Wood Technology and Renewable Resources, University of Natural Resources and Life Sciences, 3430 Tulln an der Donau, Austria

**Keywords:** drought tolerance, *Picea abies*, wood anatomy, xylem, genetic association, single nucleotide polymorphism, repeatability

## Abstract

Norway spruce (*Picea abies)* is by far the most important timber species in Europe, but its outstanding role in future forests is jeopardized by its high sensitivity to drought. We analyzed drought response of Norway spruce at the warmest fringe of its natural range. Based on a 35-year old provenance experiment we tested for genetic variation among and within seed provenances across consecutively occurring strong drought events using dendroclimatic time series. Moreover, we tested for associations between ≈1,700 variable SNPs and traits related to drought response, wood characteristics and climate-growth relationships. We found significant adaptive genetic variation among provenances originating from the species’ Alpine, Central and Southeastern European range. Genetic variation between individuals varied significantly among provenances explaining up to 44% of the phenotypic variation in drought response. Varying phenotypic correlations between drought response and wood traits confirmed differences in selection intensity among seed provenances. Significant associations were found between 29 SNPs and traits related to drought, climate-growth relationships and wood properties which explained between 11 and 43% of trait variation, though 12 of them were due to single individuals having extreme phenotypes of the respective trait. The majority of these SNPs are located within exons of genes and the most important ones are preferentially expressed in cambium and xylem expansion layers. Phenotype-genotype associations were stronger if only provenances with significant quantitative genetic variation in drought response were considered. The present study confirms the high adaptive variation of Norway spruce in Central and Southeastern Europe and demonstrates how quantitative genetic, dendroclimatic and genomic data can be linked to understand the genetic basis of adaptation to climate extremes in trees.

Norway spruce (*Picea abies* (L.) Karst.) is by far the most important timber species in Europe (Spieker 2000) and an important key species in mountainous, sub-alpine and boreal forest ecosystems. Quantitative genetic studies in Norway spruce so far mainly addressed local adaptation and trait variation at the cold end of the species’ distribution (Savolainen and Hurme 1997). Here, at its northern and altitudinal limit, temperature and photoperiodic constraints were found to result in natural selection on manifold quantitative traits (Howe *et al.* 2003), most notably bud burst, bud set, frost hardiness and growth (Skrøppa 1991; Pulkkinen 1993; Hannerz *et al.* 1999). In contrast, genetic variation and local adaptation at the warm edge of the Norway spruce range have rarely been addressed (but see Mátyás *et al.* 2009; Kapeller *et al.* 2016), although global warming is expected to reduce the distribution and plantation area of Norway spruce significantly (Hanewinkel *et al.* 2013). In particular, the species’ sensitivity to drought periods (*e.g.*, van der Maaten-Theunissen *et al.* 2013; Levesque *et al.* 2013) combined with increasing bark beetle damage (Seidl *et al.* 2008) are considered the main agents of ongoing and predicted Norway spruce decline.

Present Norway spruce populations in Central and Southeastern Europe share a complex history and originated from three to five refugial populations (Terhürne-Berson 2005; Tollefsrud *et al.* 2008). This history spans a huge range of climatic conditions including phases with little precipitation and extreme drought events, which restricted the species’ distribution during and immediately after the last glacial maximum. Today, populations at low elevations and throughout the mountainous landscape of Southeastern Europe represent the warm and dry limit of the species distribution and it is expected that this distribution edge harbors imprints of local adaptation to various climatic situations and high adaptive capacity for expected climate change scenarios (Hampe and Petit 2005; Schiessl *et al.* 2010).

Drought stress in trees may result in a variety of physiological and morphological changes: it can reduce the annual increment, can affect the xylem element architecture, may lead to alterations in the hydraulic properties, or may alter the chemical composition of the wood body (Grabner *et al.* 2006; Rosner *et al.* 2014; De Micco *et al.* 2016). Drought sensitivity is functionally related to various wood characteristics, because the vulnerability of the xylem conduits to hydraulic failure depends on conduit lumen and length as well as on cell wall thickness (Cruiziat *et al.* 2002). Therefore, wood characteristics have been suggested as screening traits for drought sensitivity to identify drought-tolerant individuals (Dalla-Salda *et al.* 2011; Rosner *et al.* 2014). However, it remains unclear whether observed trait correlations between drought sensitivity and wood characteristics (Cruiziat *et al.* 2002) are valid only within single populations or across populations and even species (George *et al.* 2015) and whether these correlations are under common genetic control. Generally, wood formation and the development of the secondary xylem constitutes a complex process that involves a combination of exogenous and endogenous factors during the different steps of cell differentiation (Fromm 2013), all of which are proposed to be coordinated by transcriptional networks (Carvalho *et al.* 2013; Zhong and Ye 2013; Liu *et al.* 2014).

Intraspecific genetic variation in drought response has been quantified in several tree species (*e.g.*, Martinez-Meier *et al.* 2008; Eilmann *et al.* 2013; George *et al.* 2015), but meaningful estimates of genetic parameters which are required for breeding activities or to assess the evolvability of populations are still rare (but see George *et al.* 2016). The predominant reason for this is that genetic testing requires controlled trials in drought-prone environments and observations over several decades in order to assess drought response on adult trees *in vivo*. Another drawback of genetic testing in field experiments is that results on small sets of genotypes and seed provenances (*i.e.*, seed material harvested from a single population) might not be transferable to other genetic backgrounds. To overcome time constraints in field tests, genome wide associations between markers and environmental conditions and/or physiological and morphological traits were proposed and applied for several tree species (Neale and Kremer 2011). Many association studies have identified SNPs and genes linked to wood and growth traits (*e.g.*, poplars [Porth *et al.* 2013], pines [Dillon *et al.* 2010] and spruces [Beaulieu *et al.* 2011; Lamara *et al.* 2016]) or to environmental variables such as aridity, drought or cold tolerance (*e.g.*, Eckert *et al.* 2010; Roschanski *et al.* 2016; Jaramillo-Correa *et al.* 2015). However, the amount of variance in quantitative traits explained by individual SNP markers is generally low and rarely exceeds 5% (Dillon *et al.* 2010; Guerra *et al.* 2013). Also, associations found in one study might not be comparable to other studies, even if they share similar sampling areas, environmental variables, and genetic loci (Ćalić *et al.* 2016). This might be explained by missing heritability of traits and insignificant local adaptation to the tested environmental gradient.

The objective of the present study was to analyze the drought response of Norway spruce in a drought-prone environment at the fringe of its natural range. Based on a 35-year old provenance experiment we tested for genetic variation among seed provenances and estimated the degree of genetic determination of the drought reaction across consecutively occurring strong drought events. Moreover, we report here the first analysis in Norway spruce which tested for genomic associations to drought reaction, wood characteristics and climate-growth relationships. The joint interpretation of the quantitative genetic analysis together with association analysis on polymorphic SNPs should enable us to identify important genes or genomic regions with a general impact on drought performance and correlated wood characteristics.

## Materials and Methods

### Trial site, plant material and sampling

We exploited data from a Norway spruce provenance trial that was established in 1978 as part of a larger test series of 44 trials in the eastern Alpine region (Nather and Holzer 1979). The trial site Porrau (48.548°N, 16.171°E) is located outside of the natural distribution area of Norway spruce at an elevation of 250 meters above sea level. Porrau is situated in the Northeast of Austria, a region characterized by high temperatures (Mean annual temperature: 9.1°), low precipitation (Annual precipitation sum: 524 mm) and frequently occurring drought periods (*e.g.*, Auer *et al.* 2005; Efthymiadis *et al.* 2006; Nobilis and Godina 2006). Seed material for the trial Porrau and the complete trial series originates from commercial seed harvests in 1971 that comprised presumably autochthonous stands and included a large number of representative seed trees (Nather and Holzer 1979; Kapeller *et al.* 2012). In addition, seed provenances from outside Austria were contributed from seed material of the IUFRO trial 1964/68 (Krutzsch 1973). The trial was established with 5-year old seedlings in a randomized block-design and plant spacing of 1.8 × 1.5 m. For the present analysis, 11 provenances (Table 1) were selected representing the distribution of the species as wide as possible and given the availability of material in the trial (Figure S1). To reveal climate-growth relationships two cores per tree were taken from 22 to 55 individuals per provenance (323 trees in total) at breast height. Due to the advanced age of the trial and unequal tree numbers within blocks, sampling and consecutive statistical analysis could not consider the original block design.

**Table 1:** Geographic and climatic origin of the analyzed provenances of *Picea abies*

Provenance and country of origin	Label	Altitude	Latitude	Longitude	MAT (°C)	APS (mm)	N
Ridelov – CZ	b03	650	49.23 N	15.4 E	6.7	717	27
Istebna – PL	d04	600	49.578 N	18.913 E	6.7	955	34
Tschepelare – BG	d14	1350 - 1500	41.736 N	24.669 E	6.2	710	30
Innsbruck – AT	I19	400 - 900	47.315 N	11.563 E	7.6	926	22
Lankowitz – AT	Q14	600	47.082 N	15.075 E	7.6	940	32
Hintergams – AT	R06	900 - 1300	47.317 N	15.203 E	3.3	1173	23
Hohenau – AT	R13	400 - 900	47.275 N	15.53 E	6.5	927	22
Klausen-Leopoldsdorf – AT	S10	500	48.078 N	16.024 E	8.4	707	24
Schneegattern – AT	ST	400 - 600	48.042 N	13.287 E	7.6	1259	55
Rosenhof-Sandl – AT	X05	800 - 850	48.582 N	14.64 E	5.5	997	27
Hartberg – AT	Y18	300 - 600	47.283 N	16.041 E	8.5	780	27

MAT-mean annual temperature, APS-annual precipitation sum, N-number of trees sampled. Labels with lower-case letters refer to provenances outside of Austria.

### Sample processing

Using a double-blade circular saw, tree cores were cut into cross sections of approximately 1.4 mm for X-ray densitometric analysis. These cross sections were placed on microfilms and exposed to a 10 kV (24 mA) X-ray source for 25 min. The films were analyzed with the software WinDENDRO 2009 (Regent Instrument, Quebec, CAN) and provided measurements of the following growth parameters: ring width (*RW*), earlywood width (*EW*, *i.e.*, wood formed in the early phase of the growing season), latewood width (*LW*, *i.e.*, wood formed late in the season consisting of narrow vessels and stronger cell walls) and latewood proportion (*LWP*), as well as the wood density characteristics; mean ring density (*RD*), earlywood density (*ED*), latewood density (*LD*), minimum density (*MIND*), and maximum density (*MAXD*) for each year of the cores. Ring width parameters were measured to the nearest 0.001 mm, while density parameters were measured in kg/m^3^. *LWP* is given in percentages as it expresses the relative proportion of latewood compared to total ring width. In order to reduce unspecific growth signals that were not caused by climatic and genetic factors (*e.g.*, eccentric tree-ring patterns caused by compression wood), values from the two cores per tree were averaged. Data were converted into single-tree time series and mean chronologies using the dplR package in R (Bunn 2010; R Development Core Team 2008).

### Identification of drought periods

For the identification of drought events, which occurred during the observation period of the trial, we used the standardized precipitation index SPI (McKee *et al.* 1993). Based on a long-term precipitation record (in our study the period 1961-2011), the SPI provides an estimate of deficit or surplus of precipitation for each point of the series. Drought events can be classified from ‘mild’ to ‘extreme’ drought events following the classification of McKee *et al.* (1993). We calculated the SPI for timescales of one and three months as these periods allowed identifying drought events with significant effects on tree growth (George *et al.* 2015). Climatic data for the trial site were interpolated from the four nearest weather stations of the Central Institute for Meteorology and Geodynamics Austria (ZAMG) using inverse distance interpolation.

### Drought response indicators

The reaction of single trees and provenances to drought events was evaluated by four measures of drought response according to Lloret *et al.* (2011): resistance, recovery, resilience and relative resilience. Resistance (*Rt*) describes how much a tree is reducing its incremental growth during drought, with *Rt* = 1 for trees which are not affected by drought, while decreasing values of *Rt* stand for increasing drought sensitivity. *Rt* is calculated as the ratio between ring-width during drought (*Dr*) and mean annual increment of the two years before the drought event (*preDr*): *Rt* = *Dr* / *preDr*. Recovery (*Rc*) is calculated as the ratio (*Rc* = *postDr* / *Dr*) of the mean annual increment across the two years following a drought year (*postDr*) and the ring-width during drought and describes how good a tree is able to recreate after a certain drought period. Higher recovery stands for faster revitalization. Resilience (*Rs*) describes the capacity of a tree to reach pre-drought increment after a drought event with *Rs* = 1 for full restoration and *Rs* < 1 indicating lasting growth reductions. *Rs* is given by the ratio of the increment of the two years after drought (*postDr*) and the pre-drought growth (*preDr*): *Rs* = *postDr* / *preDr*. By accounting for the experienced growth reduction during drought, *Rs* can be extended to the relative resilience (*rRs*) as given by *rRs* = (*postDr* - *Dr*) / *preDr*. Since a biologically caused negative long-term trend in ring width can be neglected within the relatively short time frame of each drought event and due to the equal age of the analyzed trees (Pretzsch *et al.* 2013), all drought response indicators were calculated from raw, untransformed ring width series. Also, transformations into the widely used basal area increment were unsuitable as any change of dimensionality of the measured traits affect the trait variances and thus the estimates of variance components and genetic parameters (Houle 1992).

### Climate-growth correlations

Growth dynamics of Norway spruce were found to depend on climate conditions of the local environment and the genetic origin of the seed material (Suvanto *et al.* 2016) and can be quantified by correlations between monthly climate variables and the annual increment as obtained from tree cores. For the present analysis of phenotypic trait correlations and associations to molecular markers, we calculated correlations between annual increment and monthly precipitation and temperature data for April of the preceding year to September of the current year using the entire ring chronologies. In a first step, the most important monthly climate variables were selected by testing for correlations between the mean increment across all trees as dependent variable and the monthly climate variables as independent parameter. In the second step, we removed all non-significant climate variables and calculated Pearson’s correlation coefficient between the significant climate variables and the annual increment of each individual tree and of provenance means. The obtained correlation coefficients for individual trees were then transformed to normality by using Fishers z-transformation.

### Variation among provenances

To test whether there is any genetic variation i) in drought sensitivity (*Rt*, *Rc*, *Rs*, *rRs*) within single drought periods, ii) in growth traits (*RW*, *EW*, *LW*, *LWP*) and iii) in wood characteristics (*RD*, *ED*, *LD*, *MIND*, *MAXD*), we tested for significant differences among provenances applying one-way analysis of variance (ANOVA). Here, provenances were treated as fixed effect and the respective drought, growth and wood traits as dependent variables. To confirm differences among provenances across consecutive drought periods, a repeated measure ANOVA was performed. The growth reactions of a single tree to consecutive drought events were considered as a repeated measure and treated as random effect, while provenances were treated as fixed effects. Duncan’s *post hoc* tests were used to analyze pairwise differences between provenances.

### Degree of genetic determination

To estimate the degree of genetic determination usually requires parent-offspring correlations or estimates of variance components of full- or half-sib families (Falconer and Mackay 1996). As long-term trials with such experimental designs are rarely available, we made use of the repeated occurrence of drought events at our trial site and calculated the proportion of total variation of drought response that is due to differences between individuals. This proportion is defined as repeatability (*r*) and gives the upper limit of heritability of the given trait (Boake 1989, Falconer and Mackay 1996). Repeatability is calculated as the ratio of the between-group variance $\sigma_{\alpha }^{2}$ and total phenotypic variance $\sigma_{p}^{2}$, where $\sigma_{p}^{2}$ is the sum of the between-group $\sigma_{\alpha }^{2}$ and residual (within-group) variances $\sigma_{\varepsilon }^{2}$ (Sokal and Rohlf 1995; Nakagawa and Schielzeth 2010): $r=\sigma_{\alpha }^{2}/\left(\sigma_{\alpha }^{2}+\sigma_{\varepsilon }^{2}\right)$. The variances $\sigma_{\varepsilon }^{2}$ and $\sigma_{\alpha }^{2}$ can be estimated via traditional ANOVA (Lessells and Boag 1987) or by using restricted maximum likelihood estimation (REML) in linear mixed-effect models, often referred to as the animal model (Nakagawa and Schielzeth 2010, Wilson *et al.* 2010). We used REML in the software ASReml (Gilmour *et al.* 2009) to estimate variances for two different mixed-effect models: first, we assumed equal selection pressures across all tested provenances and estimated repeatability across all provenances. Here, individual trees and provenances were treated as random effects. Second, we calculated provenance specific repeatability in univariate mixed-effect models with individual trees as single random effect. This model was applied for each provenance individually and should allow for comparing repeatability among provenances under the hypothesis that the environmental origin of the provenances has resulted in different selection pressures on drought response. For the calculation of *r*, drought response indicators were standardized and log transformed to achieve a normal-like distribution and to obtain adjusted repeatabilities (Nakagawa and Schielzeth 2010). Repeatabilities and their standard errors were calculated with the post-processing module in ASReml (Gilmour *et al.* 2009).

For phenotype-genotype associations, provenances with significant repeatabilities for drought resistance (*Rt*) were grouped into a separate subset of trees referred to as SubsetQD (Subset with significant Quantitative variation in Drought resistance). We chose drought resistance as selection parameter for the subset as resistance shows the most direct reaction of a tree to drought, while other parameters might be affected by climate conditions after drought and the trees carbon storage capacity and thus reflect more complex trait architecture.

### Phenotypic traits correlations

Drought sensitivity of trees functionally depends on xylem architecture and is related to wood characteristics. Such functional correlations indicate that wood properties, as for example wood density, might be suitable selection criteria for large-scale screening programs (Rosner *et al.* 2014) and may guide the selection of drought resistant individuals without the immediate occurrence of drought events. Thus, they are particularly beneficial in long-lived trees and field breeding experiments, where the appearance and intensity of drought events cannot be experimentally realized.

Moreover, genetic correlations are a product of local adaptation to specific environments and thus provide estimates of trait response to indirect selection (Roff 1996). As our experimental design did not allow estimating genetic correlations, we calculate phenotypic correlations, which are adequate estimates for genetic correlations if morphological and life history traits are being analyzed (Cheverud 1988; Roff 1996). We computed correlation matrices (Pearson’s product-moment-correlation) displaying the relationships between wood properties, drought stress indicators, and climate-growth relationships. Matrices were calculated across all provenances for the entire dataset (assuming equal adaptive history) as well as separately for every single provenance. Differences among provenances were expressed as minimum-maximum range for every trait combination and included in a heatmap using the package *gplot* in R (R Development Core Team 2008).

### SNP microarray design and genotyping

A custom Illumina InfiniumHD iSelect BeadChip comprising 3,257 SNPs (assays) was developed by merging SNPs from a number of different resequencing and genotyping projects. The SNP assay comprised SNPs that were identified and applied in previous studies in Norway spruce (Heuertz *et al.* 2006; Chen *et al.* 2010; Chen *et al.* 2012a, 2012b; Pavy *et al.* 2013; Källman *et al.* 2014) and related conifers (Guillet-Claude *et al.* 2004); these were also found to be useful for applications in genetic structure analysis and association studies (Chen *et al.* 2016; Heer *et al.* 2016; Ganthaler *et al.* 2017). Further details on chip design are given by Ganthaler *et al.* (2017) and a list of the compiled assays is provided in Table S1. Infinium BeadChips were manufactured by Illumina in a 24×1 format. The association analysis was done by considering 147 trees from 11 provenances (Table 1). This selection of individual trees from the total number of 323 trees was based on the phenotypic variation of drought resistance and covered extreme phenotypes with approximately 25% of trees with the highest and the 25% of tree with the lowest drought resistance. The SubsetQD further reduced the number of trees for association analysis to 72 trees by removing provenances without significant variation in drought resistance. For each sample, genomic DNA was extracted from lyophilized needle tissue using a CTAB protocol (van der Beek *et al.* 1992) with minor modifications made for the processing of 96-well plates. DNA concentrations were quantified on a 0.8% agarose gel. SNP genotyping was conducted according to the manufacturer’s recommendations and the microarray signals were detected on Illumina’s iScan System by the IMGM Laboratories GmbH, Germany. All SNP data were analyzed with GenomeStudio v. 2011.1 (Illumina).

### Population structure and relatedness

260 polymorphic neutral SNPs were used to investigate population stratification and relatedness among individuals as they can lead to false positive detection during association analysis. The selection of neutral SNPs followed a two-step procedure: first, only those SNPs located outside of genes or within gene introns were selected; second, these SNPs were filtered for a 100% call rate and a minor allele frequency (MAF) above 0.15. Population stratification was first investigated with the Bayesian model-based software STRUCTURE (Falush *et al.* 2003) which is used to infer distinct populations and to assign individuals to the identified populations. The model allows admixture and correlated allele frequencies and was run with a burn-in period of 10^5^ and Markov chain Monte Carlo (MCMC) replications after burn-in (run length) of 10^6^. Three independent runs (iterations) were conducted for each putative number of clusters (K). Sampling location information was considered by applying the prior model parameter (LOCPRIOR) to the population model (Hubisz *et al.* 2009) and possible numbers of K tested ranged from one to nine. For each scenario, Structure Harvester (Earl and von Holdt 2012) was used to estimate the most probable number of K using Evanno’s method (Evanno *et al.* 2005). Based on the first results (see Population structure), a second structure analysis was performed excluding individuals that were not related or admixed with the majority of the respective provenances, assuming that such trees may result from identification failures during seedling production or outplanting. This exclusion of trees from the total dataset increased the number of polymorphic neutral SNPs using the selection procedure above to 264 SNPs. Kinship, *i.e.*, relatedness among individuals was estimated with TASSEL´s centered IBS method using the same neutral SNPs.

### Association analysis

Associations between the different quantitative traits including drought response, wood traits, climate-growth correlations and the various SNPs was performed using TASSEL (Bradbury *et al.* 2007). SNPs with MAF less than 5% and missing genotypes higher than 10% were excluded for the association test. Overall, this resulted into a final set of 1,714 SNPs, while for the SubsetQD (*i.e.*, provenances with significant degree of genetic determination) 1,707 SNPs were tested in 72 trees. Marker-trait associations (MTAs) were calculated for all provenances and SubsetQD individually.

Association analyses were performed by using a mixed linear model taking into account population structure and kinship (MLM+Q+K) (Yu *et al.* 2006). No compression (reduction of the dimensionality of the kinship matrix to reduce computational time) was applied and variance component was re-estimated for each marker. The *p*-value threshold was corrected with the standard Bonferroni procedure resulting into the following corrected *p*-values for a given significance level α (α = ***0.001: *P* < 5.83 · 10^−7^; α = **0.01: *P* < 5.83 · 10^−6^; α = *0.05: *P* < 2.92 · 10^−5^; α = +0.1: *P* < 5.83 · 10^−5^) for all provenances and to (α = ***0.001: *P* < 5.85 · 10^−7^; α = **0.01: *P* < 5.85 · 10^−6^; α = *0.05: *P* < 2.93 · 10^−5^; α = ^+^0.1: *P* < 5.86 · 10^−5^) for SubsetQD. The amount of variation explained by a SNP was obtained for each significant association as Rsq_Marker value (R^2^). Q-Q plots were used to assess the number and magnitude of observed associations between SNPs and traits under study, compared to the association statistics expected under the null hypothesis of no association. This procedure resulted in −log10 (*p*-values) that were ranked in the order from lowest to highest on the *y*-axis and plotted against the distribution that would be expected under the null hypothesis of no association on the *x*-axis (expected values). Deviations from the identity line suggest either that the assumed population stratification or cryptic relatedness is incorrect or that the sample contains values arising from other reasons, as most likely by true associations (Wellcome Trust Case Control Consortium 2007; Pearson and Manolio 2008).

In addition to associating raw trait means to the candidate SNPs, we transformed wood property traits into binomial and categorical data (three categories). For the association study, these traits were denoted by adding b (binomial) or t (categorical) to the original trait name (ex: *RW*, *RWb*, *RWt*). Drought resistance values of the drought events (1993 and 2000) were combined into a single binomial or categorical measure by considering different scenarios as cut-off between categories (Figure S2). Drought resistance in 2003 was excluded from these combinations as individuals could be expected to show a carry-over effect from the previous drought event in the year 2000.

### Exploration of significant associations

To understand the molecular function of associated markers, we examined the Pfam domains (http://pfam.xfam.org) of the respective genes. Because of the limited quality of the available Norway spruce preliminary annotation at congenie.org, sequence similarity was assessed by BLASTP 2.6.1 (Altschul *et al.* 1997) against the GenBank NR database. SNPs located within a coding region can have a direct impact changing the amino acid sequence of a protein. Nevertheless, markers located in non-coding regions (introns, downstream regions or gene-empty regions) should also be considered due to their possible impact on gene expression through different regulatory elements as some of them could be directly involved in phenotypic variation (Webb *et al.* 2009).

All genes linked to the significantly associated SNPs were examined on the recently developed gene expression database NorWood (Jokipii-Lukkari *et al.* 2017). This resource facilitates exploration of the associated gene expression profiles and co-expression networks during wood formation and is a powerful community tool for understanding the regulation of wood development in *P. abies*.

### Data availability

The authors state that all data necessary for confirming the conclusions presented in the article are represented fully within the article. File S1 contains phenotypes for each individual. File S2 contains genotypes for each individual. File S3 contains the structure (Q) matrix used for the association study. File S4 contains the kinship (K) matrix used for the association study. File S5 contains GWAS output for all provenances. File S6 contains GWAS output for SubsetQD.

## Results

### Identification of drought periods

**Figure 1:**
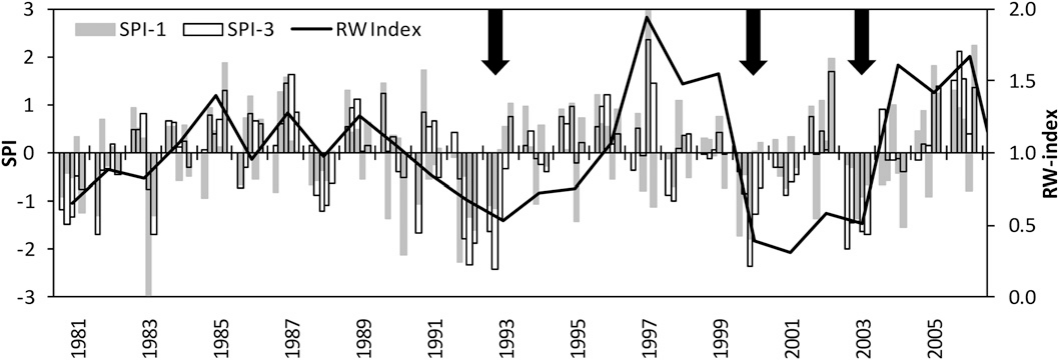
Drought occurrence and resulting increment declines of Norway spruce at a trial site located in the Northeast of Austria. The bars show the standardized precipitation index SPI (given in standard deviations) on time scales of 1 (SPI-1) and 3 (SPI-3) months. The line plots illustrate the course of annual increment, as dimensionless ring width index (RW index). Arrows mark the three most distinct drought events which effects were analyzed in the present study.

Using the SPI we identified three years (1993, 2000 and 2003) with extreme deficit of rainfall compared to the long-term record (Figure 1). These deficits were SPI=−2.41 (1993), SPI=−2.37 (2000), and SPI=−2.00 (2003) given in standard deviations according to McKee *et al.* (1993). These drought events significantly affected tree growth across all provenances. For example, the range of provenances’ growth performance (expressed as the drought stress indicator *Rs*) to the drought event in 1993 was 0.82 to 0.63, whereas it was 0.32 to 0.21 in 2000. Although the drought event in 2003 was an extreme event across Europe (Ciais *et al.* 2005), it was slightly weaker at our trial site and had smaller effects on annual increment than the two previous periods. However, the moderate growth reduction of 2003 compared to the previous two years was probably also due to the lasting effect of the previous drought in 2000. Beside these three drought events, which occurred at the beginning or in the middle of the growing season (May or June), there were also few years with SPI values of similar magnitude, but without any notable effect on tree growth (*e.g.*, 1983, 1988, and 1993; Figure 1). These further drought events were not considered for the analysis, since they occurred toward the end of the growing season (end of July, August, September; Table S2) when earlywood formation in Norway spruce was probably already completed or had at least slowed down (*e.g.*, Bouriaud *et al.* 2005).

### Climate-growth correlations

Climate-growth analysis across the complete ring chronologies of all provenances confirmed the negative effects of high temperatures on incremental growth of Norway spruce at our trial site, as we obtained significant negative correlations between annual growth and the monthly temperatures of January (*CorJanT*), March (*CorMarT*), May (*CorMayT*), June (*CorJunT*) and August (*CorAugT*) of the current year (Table S3). In contrast, high precipitation resulted in higher incremental growth as revealed by significant positive correlations to precipitation in February (*CorFebP*), May (*CorMayP*) and June (*CorJunP*). Similar correlations for individual provenances to these eight monthly climate variables confirm their importance for growth and demonstrate among-provenance variation in climate-growth response (Table S4) as correlation coefficients varied among provenances. For example, increment of provenances R6 and X5 was not correlated to precipitation in June (Table S4). Correlation coefficients between these eight monthly climate variables and ring width were calculated for each individual tree and used as traits for genetic correlation and association analysis.

### Genetic variation among provenances

**Figure 2:**
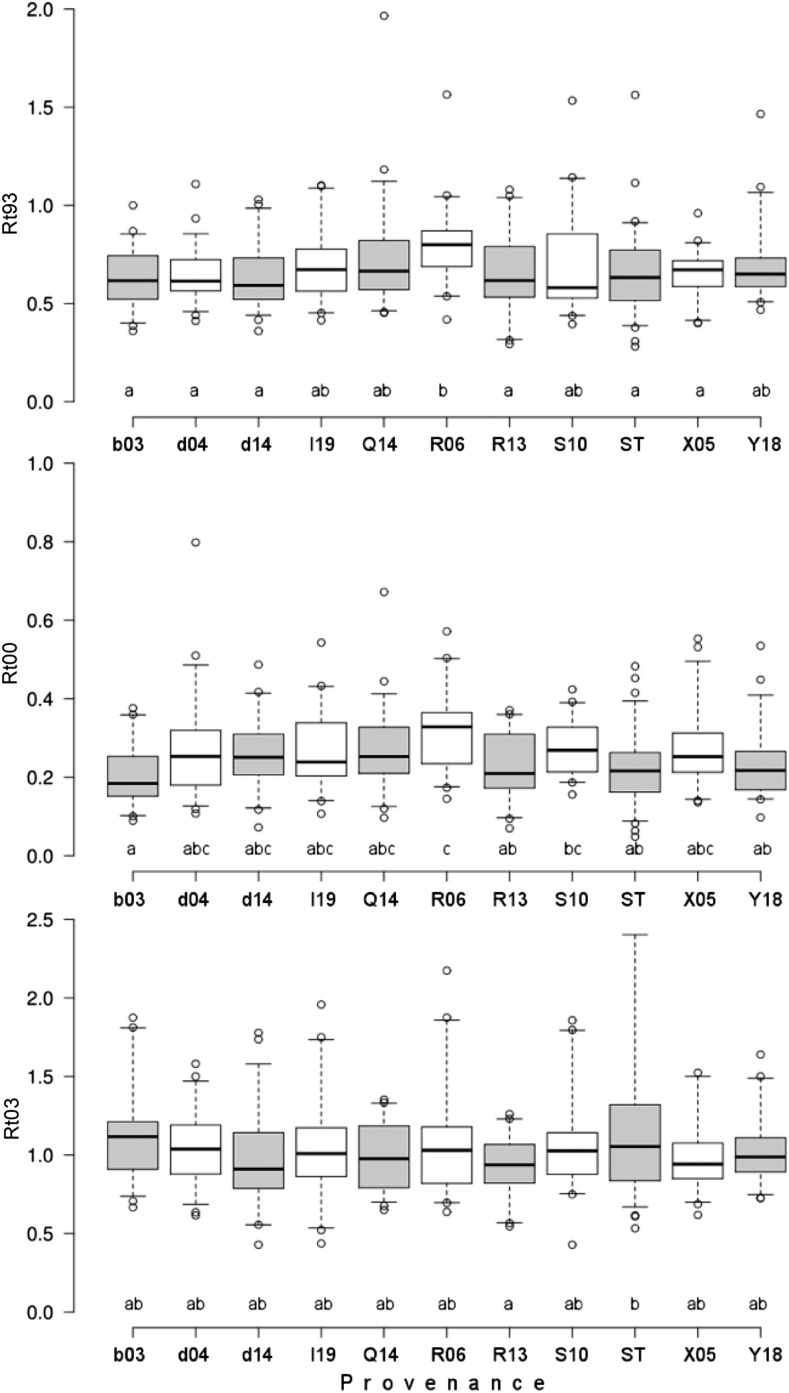
Variation in drought resistance (*Rt*) among 11 provenances of Norway spruce within the three major drought periods (1993, 2000, and 2003 – from top to bottom). Boxes gives 1^st^ to 3^rd^ quartile, band inside boxes median, and whiskers the 1.5 interquartile range. Lower case letters above the provenance label indicate result of Duncan’s multiple comparison that was applied as post-hoc test after ANOVA (Table S5).

**Figure 3:**
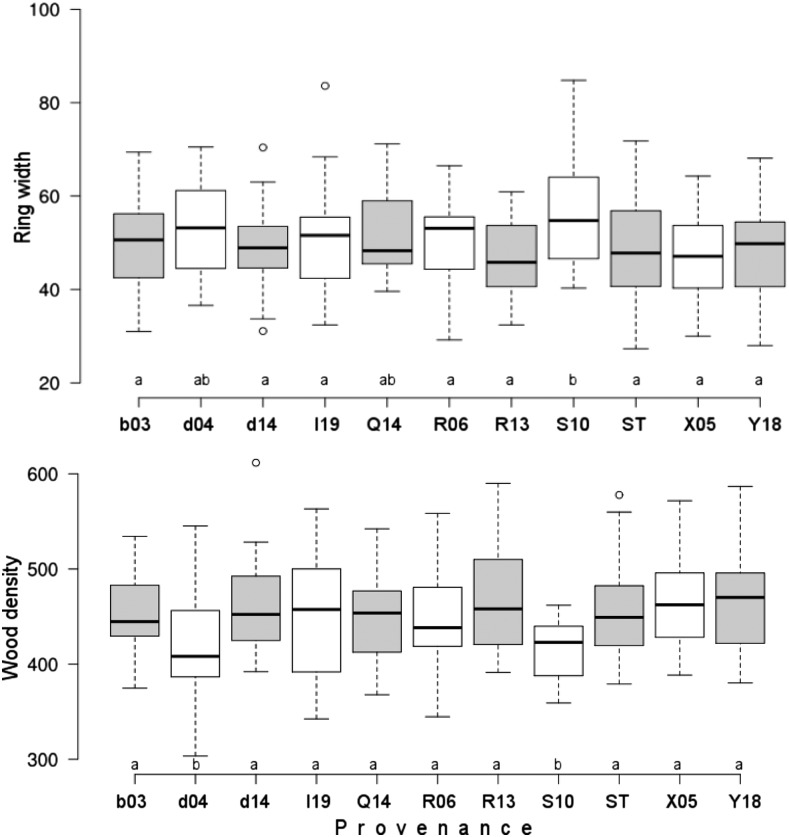
Variation in mean annual increment (ring width given in mm × 10^−2^) and mean wood density (given in g/m^3^) among 11 provenances of Norway spruce. Boxes gives 1^st^ to 3^rd^ quartile, band inside boxes median, and whiskers the 1.5 interquartile range. Lower case letters above the provenance label indicate result of Duncan’s multiple comparison that was applied as post-hoc test after ANOVA (Table S5).

Significant differences in drought response were observed among provenances for the drought events in 1993 and 2000, significant but to a lesser extent in 2003 (Figure 2, Table S5). Generally, the highest variation among provenances was observed in 2000, where also the strongest growth decline across all provenances was observed. Repeated measure ANOVA confirmed the significant differences among provenances across all three drought periods (Table S6). The best performing provenance across all drought events was R06, followed by provenances S10, Q14 and I19 (Figure 2). Significant genetic variation among provenances was also found for incremental growth and wood density parameters (Figure 3, Table S5).

### Degree of genetic determination

Treating Norway spruce as a homogenous species, the repeatability for four drought response indicators across provenances varied between nil for resilience up to 0.18 for resistance. Significant repeatabilities across all provenances were found for resistance, recovery and relative resilience (Table 2). When calculated for individual provenances, repeatabilities varied significantly between individual provenances ranging from nil to 0.44 (Table 2). The Bulgarian provenance d14 revealed significant repeatabilities for all four response measures, while the other provenances revealed significant repeatabilities only for one to three response measures. Provenances b03, d04, X05, Y18 and ST did not show significant degree of genetic determination for drought resistance *Rs* and thus were excluded from the SubsetQD (Table 2). Repeatabilities closely resemble mean pairwise correlation coefficients between drought response indicators of the individual drought periods (Table S7).

**Table 2:** Repeatabilities within each single and across all provenances as given by *r* ± SD

Provenance	Measure	Rt	Rc	Rs	rRs
b03	$\sigma_{\alpha }^{2}$	0.011 ± 0.088	0.043 ± 0.114	0.000 ± 0.000	0.053 ± 0.082
	$\sigma_{\varepsilon }^{2}$	0.737 ± 0.142	0.926 ± 0.178	0.967 ± 0.153	0.614 ± 0.118
	r	0.015 ± 0.114	0.045 ± 0.117	n.e.	0.080 ± 0.120
d04	$\sigma_{\alpha }^{2}$	0.000 ± 0.000	0.000 ± 0.000	0.000 ± 0.000	0.000 ± 0.000
	$\sigma_{\varepsilon }^{2}$	0.929 ± 0.131	0.819 ± 0.115	0.876 ± 0.123	0.893 ± 0.126
	r	0.000 ± 0.000	n.e.	0.000 ± 0.000	0.000 ± 0.000
R06*	$\sigma_{\alpha }^{2}$	0.198 ± 0.133	0.035 ± 0.077	0.000 ± 0.000	0.000 ± 0.000
	$\sigma_{\varepsilon }^{2}$	0.650 ± 0.135	0.557 ± 0.116	1.353 ± 0.232	0.813 ± 0.139
	r	**0.233 ± 0.137**	0.059 ± 0.129	0.000 ± 0.000	0.000 ± 0.000
ST	$\sigma_{\alpha }^{2}$	0.106 ± 0.110	0.404 ± 0.143	0.000 ± 0.000	0.120 ± 0.138
	$\sigma_{\varepsilon }^{2}$	1.172 ± 0.158	0.922 ± 0.124	0.912 ± 0.101	1.505 ± 0.203
	r	0.083 ± 0.084	**0.305 ± 0.088**	n.e.	0.074 ± 0.083
X05	$\sigma_{\alpha }^{2}$	0.071 ± 0.120	0.013 ± 0.078	0.000 ± 0.000	0.041 ± 0.093
	$\sigma_{\varepsilon }^{2}$	0.928 ± 0.178	0.664 ± 0.128	0.860 ± 0.136	0.734 ± 0.141
	r	0.071 ± 0.119	0.019 ± 0.115	n.e.	0.053 ± 0.118
d14*	$\sigma_{\alpha }^{2}$	0.304 ± 0.135	0.138 ± 0.092	0.162 ± 0.119	0.137 ± 0.085
	$\sigma_{\varepsilon }^{2}$	0.575 ± 0.105	0.560 ± 0.102	0.767 ± 0.140	0.498 ± 0.091
	r	**0.346 ± 0.118**	**0.198 ± 0.119**	**0.174 ± 0.119**	**0.215 ± 0.120**
I19*	$\sigma_{\alpha }^{2}$	0.151 ± 0.121	0.219 ± 0.142	0.000 ± 0.000	0.145 ± 0.110
	$\sigma_{\varepsilon }^{2}$	0.636 ± 0.136	0.648 ± 0.138	0.812 ± 0.142	0.563 ± 0.120
	r	**0.192 ± 0.140**	**0.253 ± 0.141**	n.e.	**0.205 ± 0.140**
Q14*	$\sigma_{\alpha }^{2}$	0.373 ± 0.148	0.149 ± 0.116	0.000 ± 0.000	0.022 ± 0.089
	$\sigma_{\varepsilon }^{2}$	0.584 ± 0.103	0.802 ± 0.142	1.040 ± 0.151	0.810 ± 0.143
	r	**0.389 ± 0.112**	**0.157 ± 0.114**	n.e.	0.027 ± 0.106
R13*	$\sigma_{\alpha }^{2}$	0.517 ± 0.232	0.162 ± 0.216	0.000 ± 0.000	0.000 ± 0.000
	$\sigma_{\varepsilon }^{2}$	0.657 ± 0.140	1.388 ± 0.296	1.284 ± 0.225	1.217 ± 0.214
	r	**0.440 ± 0.132**	0.105 ± 0.135	0.000 ± 0.000	0.000 ± 0.000
S10*	$\sigma_{\alpha }^{2}$	0.381 ± 0.171	0.239 ± 0.124	0.000 ± 0.000	0.083 ± 0.087
	$\sigma_{\varepsilon }^{2}$	0.552 ± 0.113	0.498 ± 0.102	1.012 ± 0.170	0.550 ± 0.112
	r	**0.408 ± 0.129**	**0.324 ± 0.133**	n.e.	**0.131 ± 0.131**
Y18	$\sigma_{\alpha }^{2}$	0.061 ± 0.112	0.000 ± 0.000	0.000 ± 0.000	0.000 ± 0.000
	$\sigma_{\varepsilon }^{2}$	0.872 ± 0.168	1.155 ± 0.183	0.957 ± 0.151	1.381 ± 0.219
	r	0.065 ± 0.119	n.e.	n.e.	0.000 ± 0.000
Overall	$\sigma_{pro}^{2}$	0.022 ± 0.018	0.032 ± 0.021	0.016 ± 0.012	0.014 ± 0.012
	$\sigma_{\alpha }^{2}$	0.182 ± 0.039	0.129 ± 0.036	0.000 ± 0.000	0.037 ± 0.033
	$\sigma_{\varepsilon }^{2}$	0.798 ± 0.044	0.838 ± 0.047	0.983 ± 0.045	0.947 ± 0.053
	r	**0.182 ± 0.036**	**0.129 ± 0.035**	0.000 ± 0.000	**0.037 ± 0.033**

* Provenances that were grouped into the SubsetQD for additional association analysis.

$\sigma_{pro}^{2}$ - Variance among provenances; $\sigma_{\varepsilon }^{2}$ – Variance within groups; $\sigma_{\alpha }^{2}$ – variance among groups. Boldface indicates significant repeatabilities.

### Phenotypic trait correlations

**Figure 4:**
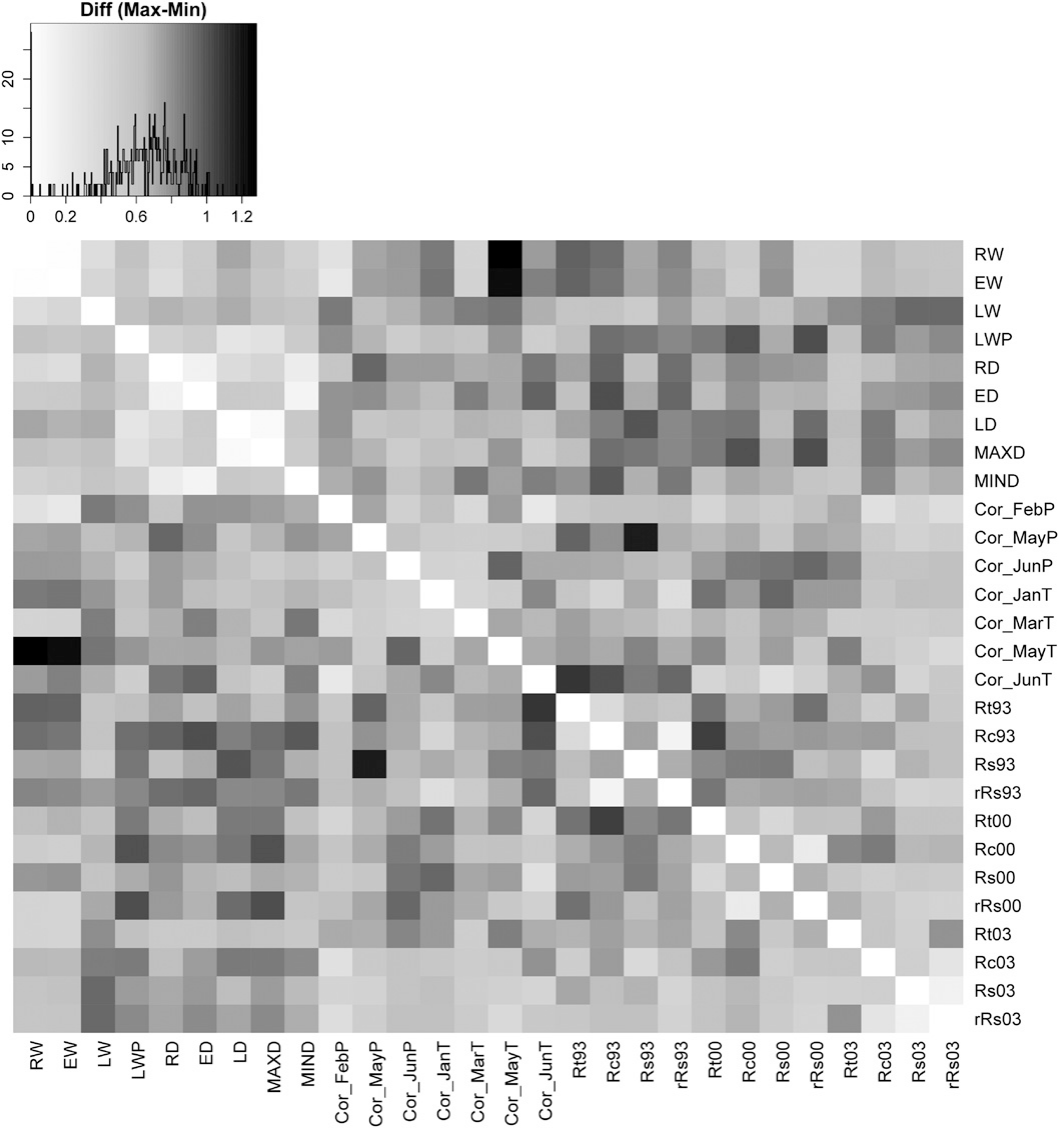
Variation in trait-trait correlations among provenances. Shown are max-min differences. For example, the correlation between resilience during the drought in 1993 (*Rs93*) and growth response to May precipitation (*CorMayP*) was 0.62 for provenance B3 whereas it was -0.55 for provenance Q14 (both significant at *P* < 0.001 and <0.01, respectively; Figure 5). The color key in the upper left corner shows the absolute correlation difference (x-axis) as well as the frequency of occurrence (black line on y-axis). See also Figure 5 for two explanatory examples.

**Figure 5:**
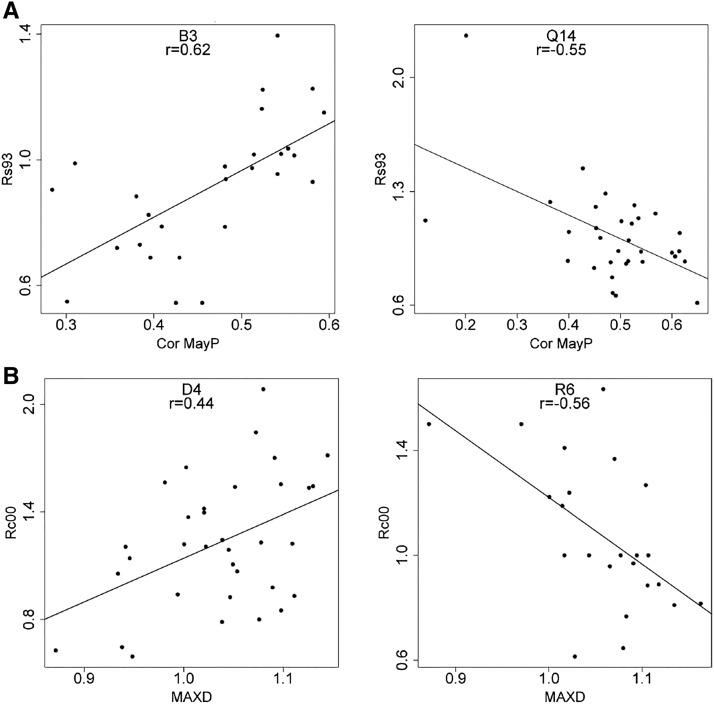
Variation in phenotypic correlations among provenances for two selected trait combinations. (A) Correlation between resilience in 1993 and growth response to May precipitation for two different provenances. (B) Correlation between recovery and maximum density for two different provenances.

Genetic and phenotypic trait correlations are important indicators for indirect selection on specific traits and could guide the development of screening tools for drought-resistant genotypes in breeding programs. When we tested for provenance-specific differences in the complex relationships between wood properties, drought stress indicators, and climate-growth correlations, the correlation matrices revealed ample differences among provenances for numerous trait combinations (Figure 4). Generally, variation in trait correlations among wood properties, among drought indicators and among climate-growth correlations were small, but correlations between drought indicators and wood properties or between drought indicators and climate-growth correlations were high and ranged from positive correlations in one provenance to negative correlations within another. For example, the correlation between resilience during the drought in 1993 (*Rs93*) and growth response to May precipitation (*CorMayP*) was 0.62 for provenance B3 whereas it was −0.55 for provenance Q14 (both significant at *P* < 0.001 and <0.01, respectively; Figure 5). When we tested for correlations across all provenances (*i.e.*, for the entire dataset), the same correlations were rather weak or even non-significant, justifying provenance specific trait correlations (Figure S3).

### Population structure

To avoid spurious false positive associations between SNPs and quantitative traits, we tested for population structure and relatedness with 260 polymorphic neutral SNPs. When considering 155 trees from eleven provenances in population structure analysis, the likelihood (LnP) of K increased reaching a continuous plateau with large variances (Figure S4A). Using Evanno’s method, the Delta K plot showed low values in all Ks that were tested for, but two peaks for K = 4 and K = 7 with the latter one being the highest (Figure S4A). Bar plots with 155 individuals illustrated the uncertain assignment of individuals to the seven groups. In particular, six individuals from provenance S10 and one individual each of d04 and b03 showed genotypic information that was very distinct from all analyzed provenances (Figure S4A). After these eight genotypes were removed and the structure analysis recalculated with 147 individuals from eleven provenances the LnP of K increased reaching a continuous plateau with reasonably small variances (Figure S4B). Evanno’s method resulted into a highest Delta K for K = 2 as illustrated by bar plots of the 147 individuals (Figure S4B). This structure result (Q) was subsequently used for all association analyses. Kinship estimated for these 147 individuals resulted in a matrix with values ranging mainly from 0.1 to −0.1 and a slightly negative mean kinship value of −0.007 (Figure S5).

### Association analysis

**Figure 6:**
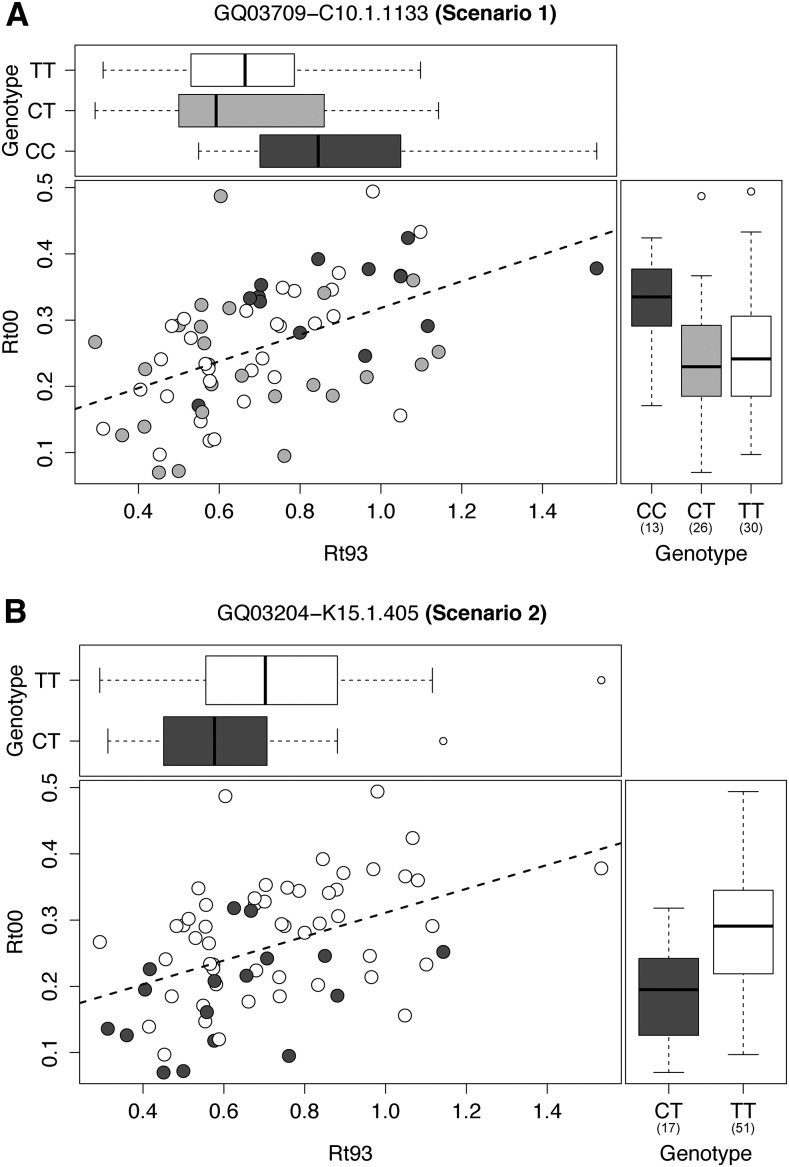
(A) Scatterplot of *Rt93 vs. Rt00* together with marginal boxplots of genotypes from marker GQ03709-C10.1.1133 associated to Scenario 1; Genotypes were colored as follows: dark gray (CC), gray (CT) and white (TT). (B) Scatterplot of *Rt93 vs. Rt00* together with marginal boxplots of genotypes from marker GQ03204-K15.1.405 associated to Scenario 2; Genotypes were colored as follows: white (TT) and dark gray (CT). Dashed lines represent linear regression and boxes within the marginal boxplots represent the median (black middle line) limited by the 25^th^ (Q1) and 75^th^ (Q3). Numbers below each genotype indicates sample sizes.

147 trees from 11 provenances (Table 1) were considered for the association analysis (Figure S6). First, all provenances were included in the association study. Based on the quantitative genetic analysis a second analysis was performed with a subset of provenances that revealed significant quantitative genetic variation within provenances, *i.e.*, significant degree of genetic determination (SubsetQD with provenances R06, d14, I19, Q14, R13 and S10). With the MLM+Q+K model, 1,714 SNPs (all provenances) and 1,707 SNPs (SubsetQD) were tested against 51 traits: four drought scenario traits (*Scen1*, *Scen2*, *Scen3*, *Scen4*), 12 drought stress parameters (*Rt93*, *Rc93*, *Rs93*, *rRs93*, *Rt00*, *Rc00*, *Rs00*, *rRs00*, *Rt03*, *Rc03*, *Rs03*, *rRs03*), eight climate-growth correlations (*CorFebP*, *CorMayP*, *CorJunP*, *CorJanT*, *CorMarT*, *CorMayT*, *CorJunT*, *CorAugT*) and 27 wood property traits (*RW*, *EW*, *LW*, *LWP*, *RD*, *ED*, *LD*, *MAXD*, *MIND*, +b [binomial], +t [categorical]). Among 87,414 marker-trait pairs 17 significant associations were found for all provenances. For the SubsetQD, we found 20 significant associations (Table 3, Figure 6, Figure S7) among the 87,057 marker-trait pairs. As some SNPs were associated with several correlated traits and some SNPs were significant in both sets, a total of 29 SNPs—physically located within or in close neighborhood of 24 genes—were found to be associated with the studied traits (Table 4). Significant SNPs found in both sets (all provenances and SubsetQD) revealed genes and genotypes that are linked to drought response and wood quality across the complete Central European range of Norway spruce, while those markers found to be significant within the SubsetQD might indicate local adaptations to drought prone environments. Among all SNPs detected, 12 were mainly detected in single individuals having extreme phenotypes of the respective trait (Table 3).

**Table 3:** Significant genetic associations

Category	Trait		Marker		ALL PROVENANCES			SUBSETQD		
					p-value		R^2^	p-value		R^2^
Scenarios	Scen1		GQ03709-C10.1.1133					1.55E-05	*	0.33
	Scen2		GQ03204-K15.1.405					5.69E-05	^+^	0.36
Drought-2000	Rc00		ss538948434					1.30E-05	*	0.29
		^a^	c89584_g2_i1_197		2.28E-05	*	0.14			
			PGLM2-0082		2.87E-05	*	0.14			
	Rs00		WS-2.0-GQ02827.B7-M03.1-1062		1.90E-06	**	0.15	6.63E-06	*	0.26
			NODE-12228-length-276-cov-134.847824-162		1.08E-05	*	0.15			
		^b^	WS-2.0-GQ03417.B7-O19.1-1215	S	1.34E-05	*	0.15			
	rRs00		GQ03812-F08.1.123	S				2.54E-07	***	0.36
		^b^	WS-2.0-GQ03417.B7-O19.1-1215	S				1.34E-05	*	0.29
Drought-2003	Rt03	^c^	MA_475589g0010-111-[C_T]	S	2.25E-11	***	0.31			
			ss538948838	S	3.64E-07	***	0.19			
		^a^	c89584_g2_i1_197		1.42E-05	*	0.14			
			GQ03512-P11.2.1194		3.45E-05	^+^	0.13			
			GQ03013-D24.3.551		5.74E-05	^+^	0.13			
	Rc03		ss538944271					4.78E-05	^+^	0.26
			GQ0197-K07.1.447	S	1.91E-06	**	0.17			
			GQ0197-K07.1.345	S	1.91E-06	**	0.17			
			GQ0068-K02.1.644	S	1.58E-05	*	0.14			
			GQ02904-L21.2.961		3.76E-05	^+^	0.13			
	Rs03	^c^	MA_475589g0010-111-[C_T]	S	1.81E-05	*	0.15			
Climate-growth	CorJunT		GQ03204-O22.1.645					1.91E-05	*	0.25
	CorJunT		FCL808Contig2-402					3.09E-05	^+^	0.24
	CorJunT		MA_10289324g0010-1023-[G_A]					4.26E-05	^+^	0.23
Wood Properties	RW	^d^	08Pg07115c					3.23E-06	**	0.29
	RWt	^d^	08Pg07115c					3.98E-06	**	0.28
	EW	^d^	08Pg07115c					5.19E-06	**	0.27
	LW		GQ03709-C10.1.483					7.97E-09	***	0.42
			GQ03121-E24.1.494	S				9.84E-09	***	0.42
			PaPHYN-RI420	S				1.54E-08	***	0.41
			GQ03205-D16.1.1410	S				1.73E-08	***	0.41
			GQ00410.B3-M23.4-2998	S	1.12E-08	***	0.24	2.64E-08	***	0.43
	RD	^e^	WS-2.0-GQ0036.TB-K03.1-397					5.32E-05	^+^	0.25
	RDt	^e^	WS-2.0-GQ0036.TB-K03.1-397					2.43E-05	*	0.26
	EDt		00930-O17-366		4.43E-05	^+^	0.11			

The p-value threshold was corrected with the standard Bonferroni procedure resulting into the following corrected p-values for a given significance level ⍺: All provenances (⍺ = ***0.001: *P* < 5.83 · 10-7; ⍺ = **0.01: *P* < 5.83 · 10-6; ⍺ = *0.05: *P* < 2.92 · 10-5; ⍺ = ^+^0.1: *P* < 5.83 · 10-5); SubsetQD (⍺ = ***0.001: *P* < 5.85 · 10-7; ⍺ = **0.01: *P* < 5.85 · 10-6; ⍺ = *0.05: *P* < 2.93 · 10-5; ⍺ = ^+^0.1: *P* < 5.86 · 10-5).

a,b,c,d,e SNP significantly associated to several traits.

S: SNP significantly associated because of a single individual with an extreme trait value.

**Table 4:** Characteristics of markers significantly associated

Marker name	Associated Trait	Genomic position	Nucleotide	Exon / Intron	Strand	SNP	SNP type*^a^*	Codon position	Non synonymous substitution / Missense change	PFAM Description	BLAST-P *^b^*
GQ03709-C10.1.1133	Scen1	No gene - downstream (13 nt) MA_59794g0010	21494		+	[T/C]	UTR			*	Unknown
GQ03204-K15.1.405	Scen2	No gene - upstream (164 nt) MA_10434266g0010	3694		—	[T/C]	UTR			Leucine Rich Repeat	No significant similarity found.
08Pg07115c	RW; RWt; EW	No gene - downstream (297 nt) MA_39636g0010	24594		—	[T/C]	UTR			Transferase family	Hydroxycinnamoyl-CoA shikimate hydroxycinnamoyltransferase
GQ03709-C10.1.483	LW	MA_59794g0010	20695	Exon 1	+	[T/C]	NSS-M	2	GCA = A (Alanine) / GTA = V (Valine)	*	Unknown
GQ03121-E24.1.494	LW	MA_6741659g0010	1238	Exon 2	+	[T/C]	SS	3	CG[T/C]=R (Arginine)	*	Kinase
PaPHYN-RI420	LW	MA_73153g0010	13476	Exon 6	—	[A/C]	NSS-M	3	GAA=E (Glutamic acid)	Phytochrome region	Phytochrome
									GAC=D (Aspartic acid)	PAS fold	
										PAS domain	
										GAF domain	
										Histidine kinase	
										DNA gyrase B	
										HSP90-like ATPase	
GQ03205-D16.1.1410	LW	MA_10432182g0010	2114	Intron	—	[A/C]	UTR			Short chain dehydrogenase	Short chain dehydrogenase
										NAD dependent epimerase/dehydratase family	
										NmrA-like family	
										KR domain	
										NADH(P)-binding	
										Enoyl-(Acyl carrier protein) reductase	
GQ00410.B3-M23.4-2998	LW	MA_17539g0010	1887	Exon 1	+	[A/G] §	SS	3	GA[T/C]=D (Aspartic acid)	EF hand / EF-hand domain pair	Calcium-binding protein
WS-2.0-GQ0036.TB-K03.1-397	RD;RDt	MA_10426694g0020	18480	Exon 3	—	[T/C] §	SS	3	GG[A/G]=G (Glycine)	Metallopeptidase family M24	ERBB-3 BINDING PROTEIN 1
00930-O17-366	EDt	MA_588952g0010	731	Exon 1	—	[A/C]	SS	3	CT[C/A]=L (Leucine)	*	No significant similarity found.
ss538948434	Rc00	MA_100637g0010	3240	Exon 3	+	[A/G]	NSS-M	1	GTT = V (Valine)	PPR repeat	Pentatricopeptide repeat-containing protein
									ATT = I (Isoleucine)	Herpesvirus protein of unknown function (DUF832)	
										Tetratricopeptide repeat	
										Anaphase-promoting complex, cyclosome, subunit 3	
										PPR repeat family	
										Pentatricopeptide repeat domain	
c89584_g2_i1_197	Rc00;Rt03	No gene - downstream (418 nt) MA_90007g0010	15514		—	[A/C]	UTR			BTB/POZ domain	ARM repeat protein interacting with ABF2
PGLM2-0082	Rc00	MA_8047450g0010	2537	Exon 2	—	[A/G] §	SS	3	CA[T/C]=H (Histidine)	RNA recognition motif. (a.k.a. RRM, RBD, or RNP domain)	Unknown
										ROKNT (NUC014) domain	
WS-2.0-GQ02827.B7-M03.1-1062	Rs00	No gene (MA_75479)	904		+	[A/G]	UTR				
NODE-12228-length-276-cov-134.847824-162	Rs00	Not in 1kb MA_118377	17768		—	[T/C]	UTR				
WS-2.0-GQ03417.B7-O19.1-1215	Rs00,rRs00	No gene - downstream (104 nt) MA_119424g0010	10110		—	[T/G]	UTR			Short chain dehydrogenase	Anthocyanidin reductase
										3-beta hydroxysteroid dehydrogenase/isomerase family	
										NAD dependent epimerase/dehydratase family	
										NmrA-like family	
										Male sterility protein	
										KR domain	
										NADH(P)-binding	
GQ03812-F08.1.123	Rs00;rRs00	No gene (MA_7880)	20633		—	[A/G]	UTR				
MA_475589g0010-111-[C_T]	Rs03;Rt03	MA_475589g0010	5299	Exon 1	—	[T/C]	SS	3	GG[T/C]=G (Glicine)	Leucine Rich Repeat	F-box/LRR-repeat protein
ss538948838	Rt03	MA_109428g0010	15103	Exon 4	+	[T/G]	NSS-M	1	TCA = S (Serine)	Translocon-associated protein (TRAP), alpha subunit	Translocon-associated protein subunit alpha
									GCA = A (Alanine)	NICE-3 protein	
GQ03512-P11.2.1194	Rt03	MA_10427719g0010	15051	Intron	—	[T/G]	UTR			2OG-Fe(II) oxygenase superfamily	
										Non-haem dioxygenase in morphine synthesis N-terminal	
GQ03013-D24.3.551	Rt03	MA_10434370g0040	13176	Exon 2	—	[T/C]	SS	3	GA[T/C]=D (Aspartic acid)	*	EGF domain-specific O-linked N-acetylglucosamine transferase-like
ss538944271	Rc03	MA_161013g0010	7889	Intron	+	[A/G]	UTR			Multicopper oxidase	Multicopper oxidase
											L-ascorbate oxidase homolog
GQ0197-K07.1.447	Rc03	MA_38472g0010	6199	Exon 6	—	[T/C]	SS	3	AA[T/C]=D (Aspartic acid)	Homeobox domain	Homeodomain protein HB2
GQ0197-K07.1.345	Rc03	MA_38472g0010	6301	Exon 6	—	[A/G]	SS	3	GG[A/G]=G (Glicine)	START domain	Homeobox-leucine zipper protein ANTHOCYANINLESS 2-like isoform
										Homeobox KN domain	
										Mitochondrial ribosomal protein subunit L20	
GQ0068-K02.1.644	Rc03	MA_47260g0010	3906	Exon 4	—	[T/C]	SS	3	TC[T/C]=S (Serine)	Tetratricopeptide repeat	Clathrin heavy chain 1
										Region in Clathrin and VPS	
										Coatomer WD associated region	
										Clathrin-H-link	
GQ02904-L21.2.961	Rc03	MA_10432150g0010	33483	Exon 2	+	[T/C]	NSS-M	2	CCG = P (Proline)	WD domain, G-beta repeat	WD repeat-containing protein VIP3
									CTG = L (Leucine)	Cytochrome D1 heme domain	
										Coatomer WD associated region	
										Eukaryotic translation initiation factor eIF2A	
										Lactonase, 7-bladed beta-propeller	
										Nucleoporin Nup120/160	
GQ03204-O22.1.645	CorJunT	MA_6521216g0010	1742	Exon 1	—	[A/G]	SS	3	GA[A/G]= E (Glutamic acid)	*	Dihydropyrimidinase
FCL808Contig2-402	CorJunT	MA_2267030g0010	105	Exon 1	+	[A/G]	SS	3	GT[A/G]=V (Valine)	CutA1 divalent ion tolerance protein	Protein CutA
MA_10289324g0010-1023-[G_A]	CorJunT	MA_10289324g0010	1649	Exon 2	+	[A/G]	SS	3	CT[A/G]=L (Leucine)	2OG-Fe(II) oxygenase superfamily	GA2OX3 (Gibberellin 2-beta-dioxygenase 3)
										Non-haem dioxygenase in morphine synthesis N-terminal	

a SS= Synonymous substitution; NSS-M= non synonymous substitution Missense change, UTR= untranslated regions.

b Putative gene function assigned by BLAST-P (Table S4 for complete BLAST-P output).

* PFAM ID could not be found.

§ SNP on the complementary strand of a translated protein.

In general, the amount of variation explained by associated SNPs was higher for SubsetQD (R^2^ = 0.23-0.43) compared to all provenances (R^2^ = 0.11-0.31). For SubsetQD, markers GQ03709-C10.1.1133 (R^2^ = 0.33) and GQ03204-K15.1.405 (R^2^ = 0.36) are significantly associated to drought resistance scenario one and two which combine continuous drought resistance in 1993 and 2000 into a single binomial trait (Figure 6, Figure S2). For GQ03204-K15.1.405, homozygous TT genotypes had higher drought resistance than heterozygous CT. On the other hand, homozygous CC genotypes of GQ03709-C10.1.1133 were found to have higher drought resistance than TT or CT genotypes. From the continuous drought traits, associations were only found for response variables in the periods 2000 and 2003. Traits of the drought event 2000 (*Rc00*, *Rs00* and *rRs00*) were associated to four SNPs with R^2^ between 0.26 and 0.36, while from the drought 2003 only a single response variable (*Rc03*) was associated to a SNP (ss538944271) with R^2^ = 0.26. Finally, three SNPs with R^2^ between 0.23 and 0.25 were found to be associated to the correlation between June temperature and annual ring width (*CorJunT)*: GQ03204-O22.1.645, FCL808Contig2-402, MA_10289324g0010-1023-[G_A].

Significant genotype-trait associations for 7 SNPs were also found for wood property traits (*RW*, *EW*, *LW*, *RD*, *RDt*, *RWt*, *EDt*) showing the highest R^2^ (between 0.28 and 0.43) within our study. Among them, SNP 08Pg07115c was significantly associated to both *RW* and *EW* (R^2^ = 0.27-0.29). SNP GQ03709-C10.1.483 which is associated to *LW* (R^2^ = 0.42) is located 799 nucleotides upstream of GQ03709-C10.1.1133, *i.e.*, one of the SNPs associated with drought response. Also, a single association to wood density (*RD*, *RDt*) was detected (WS-2.0-GQ0036.TB-K03.1-397, R^2^ = 0.25-0.26).

When all provenances were considered for the association study, no single SNP was found to be associated with a scenario of drought resistance. However, several significant associations were found for drought response indicators for the drought events in 2000 and 2003. Traits of the drought 2000 (*Rc00*, *Rs00*) were associated to five SNPs (R^2^ = 0.14-0.15), with one of them (WS-2.0-GQ02827.B7-M03.1-1062) also detected within the SubsetQD. For 2003, ten SNPs were associated to three traits (*Rt03*, *Rc03* and *Rs03*). In few cases, SNPs were associated to different drought traits of the same drought event, but also across different drought events. Two SNPs were significantly associated with wood properties: 00930-O17-366 was associated to ED (R^2^ = 0.11) and GQ00410.B3-M23.4-2998, also detected within the SubsetQD, to LW (R^2^ = 0.24).

SNPs significantly associated were mainly located within exons (18 SNPs). Others (11 SNPs) belonged to untranslated regions (UTR) like introns, downstream regions or gene-empty regions (Table 4). Variations in five exon-located SNPs were found to affect the amino acid sequence (non-synonymous substitution; missense change). On the other hand, variations in the remaining 13 exon-located SNPs were found to have no effect on the amino acid sequence (synonymous substitution) as they are located mainly in the 3^rd^ codon position.

### Exploration of associated genes

Exploration of the 24 genes in which the 29 SNPs are located or to which they were closely linked indicates a highly diverse assortment of Pfam domains (Table 4). Additionally, BLASTP analysis (Table S8) allowed annotations of some genes (*e.g.*, MA_39636g0010: Putative hydroxycinnamoyl-CoA shikimate/quinate hydroxycinnamoyl transferase involved in lignin biosynthesis pathway; MA_90007g0010: ARM repeat protein interacting with ABF2) but did not reveal similarity to any known genes for some others (*e.g.*, MA_10434266g0010; MA_588952g0010).

Exploration of the expression profiles of the associated marker genes within the gene expression resource NorWood (Figure S8) resulted in expression data for 20 out of the 24 associated genes. Hierarchical clustering of these expression data revealed seven clusters (Figure S8A). Out of these, one cluster (cluster-c) showed a well defined expression profile across the stem section (Figure S8B) with maximum expression within cambium (T-03, T-04) and xylem expansion cells (T-05, T-06). Cluster-c comprised 5 genes including multicopper oxidase / L-ascorbate oxidase, Leucine Rich Repeat (LRR) and ERBB-3 binding protein 1; Table 4). Other gene clusters have maximum expression within the zone of secondary wall formation (cluster-a), or within mature xylem (cluster-e).

## Discussion

Under global warming, extreme climate events are expected to increase in frequency, duration and intensity and such events are considered the main causes for forest mortality and dieback (Allen *et al.* 2010). In this study, we analyzed the genetic variation of drought response of Norway spruce by means of quantitative and molecular genetic analysis of specimens growing for 35 years in a drought-prone environment. During this time, three strong drought events resulted in growth reductions of up to 80% revealing significant genetic variation among and within the tested provenances. On basis of the observed quantitative genetic variation, we found significant associations between drought response indicators and wood properties for a total of 29 SNPs, though 12 of them were due to single individuals having extreme phenotypes of the respective trait. These included SNPs closely linked to genes involved in lignin biosynthesis, cell differentiation or transcription. Our study is one of the first that links dendroclimatic analysis, quantitative genetic provenance and breeding research with genomic association studies. By making use of existing trials that experienced *in situ* stress events that were archived in tree rings, we were able to provide meaningful phenotypes for the association analysis. Moreover, restricting association analysis to provenances with significant quantitative genetic variation among individuals resulted in stronger and likely more meaningful phenotype-genotype associations.

### Quantitative genetic variation

Among various European conifers, Norway spruce was found to show the highest vulnerability to extreme heat and drought events (Lévesque *et al.* 2013; Zang *et al.* 2014). Thus, it is remarkable that we observed significant variation in drought response among as well as within some of the tested provenances. However, this can be explained by the broad geographic amplitude of the tested plant material, which comprised provenances of the Eastern Alpine range, the western Carpathian Mountains, the Bohemian Massive and the Rhodope Mountains at the southern fringe of the species. This geographic area harbors high plastid diversity as a consequence of multiple refugial areas and postglacial migration history as confirmed by previous genetic studies (Collignon *et al.* 2002; Tollefsrud *et al.* 2008; Mengl *et al.* 2009) and as expected at the rear edge of the species’ range (Hampe and Petit 2005). However, neutral genetic diversity in nuclear genes within the Alpine and Central European domain was found to be relatively low in Norway spruce (Heuertz *et al.* 2006) as we could confirm in our analysis with 264 neutral SNPs. These markers show only a low differentiation with a global *F_st_* of only 0.015, probably resulting from extensive gene flow via pollen which is able to mediate effective gene transfer even between refugial lineages (Liepelt *et al.* 2002) On the other hand, analysis of the 29 SNPs which are associated to drought and wood traits revealed a higher global *F_st_* of 0.045 and thus indicates slightly higher differentiation at markers which are likely to be under selection.

Generally, studies on the genetic variation of drought response in Norway spruce are rare. Burczyk and Giertych (1991) analyzed growth reaction of 17 polish provenances within drought years in the 1980s. In contrast to our study, Burczyk and Giertych (1991) did not find differences among provenances, they just observed a weak genotype x environment interaction and concluded that selection for drought resistance in Norway spruce might not be successful. This conclusion may rely on the tested genetic material, which consisted only of provenances from Poland and thus represented a small and genetically homogeneous part of the natural range. Actually, the single Polish provenance in our test, d04-Istebna (similar to Prov. 99 in Burczyk and Giertych 1991) neither showed remarkable drought response nor revealed significant repeatability for any of the applied drought measures. This indicates that selection pressures for drought might be rather weak and the response of trees therefore uniform within the northwestern Carpathian Mountains. More consenting evidence of genetic variation in drought response comes from several seedling studies. Schmidt-Vogt (1978) tested drought resistance in seedlings of five Central European provenances and obtained significant differences among the seedling origins. Here, provenances from higher elevations were found to show higher suction potential and this was interpreted as better adaptation to water-limited conditions (Schmidt-Vogt 1978). More recently, this finding was confirmed (Blödner *et al.* 2005) in one-year old seedlings by investigating physiological traits related to drought and freezing sensitivity. Blödner *et al.* (2005) found drought-sensitivity and freezing-sensitivity to be co-occurring traits suggesting that the physiological reactions for both environmental extremes are based on comparable oxidative stress protection systems.

Evidence for genetic variation in drought resistance within populations comes from a seedling experiment with 12 clones originating from another single Alpine population (Wagner 1993). Wagner (1993, 1994) measured stress respiration, photosynthesis, needle morphology and drought tolerance on clonal propagules from 12 Alpine trees and found significant variation among clones as well as correlations between the physiological traits and growth performance at tree age of 12. Similar inter-clonal variation of hydraulic properties was found within young and mature trunk wood of 5- and 24-year old Norway spruce trials consisting of vegetatively propagated clonal material (Rosner *et al.* 2007, 2008). Broad sense heritability for measures of hydraulic conductivity within these studies ranged from 0.14 to 0.31 for the different conductivity parameters (Rosner *et al.* 2007). Higher broad sense heritabilities between 0.10 and 0.41 were found for wood density peaks initiated by strong water deficits in clonal trials in southern Sweden (Rozenberg *et al.* 2002). Our study confirms the genetic variation in drought response among individuals within and among provenances. The highest repeatabilities were found for resistance, slightly smaller ones for recovery, resilience and relative resilience. These differences can likely be explained by the complexity of physiological processes underlying the different dendrologically-based drought measures. Resistance captures the ultimate response of a tree to an ongoing drought and is thus the most direct parameter of drought response. The remaining parameters include the recovery period and are likely affected by climate conditions after drought or the individual allocation of non-structural carbohydrates (NSC). This complex trait architecture of drought response makes quantitative genetic studies as well as genotype-trait associations difficult.

Besides differences among the drought response indicators, we were able to demonstrate that some provenances exhibit high degrees of genetic determination for one or more of the calculated drought indicators, while others do not reflect any repeatability. For example, provenance d14 from high elevations at the southern fringe of the species range revealed high repeatability of up to 0.35 for all four drought indicators. Although our data do not reveal a clear climatic or geographic trend to drought response or genetic determination, they strongly indicate varying selection pressures within the seed source populations. This argument is strongly supported by highly variable phenotypic correlations between drought response indicators and morphological traits (Cheverud 1988; Roff 1996). When comparing phenotypic trait correlations among provenances, we observed strongly varying patterns, which differ not only in magnitude, but also in direction (Figures 4 and 5). Generally, theoretical studies and laboratory analysis indicate that correlations between life history traits (and we may consider drought response as such) should rather be negative and correlations among life history traits are typically larger than between life history traits and morphological traits (Roff 1996). However, when tested across different environments, genetic correlations might change from negative to positive even when estimated on the same genetic material (Sgrò and Hoffmann 2004). In this context, our variable correlation patterns might be explained by differences in the genetic system, *i.e.*, the extent of pleiotropy, the demographic history of the provenances or due to differences in selection intensity (Lascoux 1997).

Across provenances, drought resistance (in 1993 and 2000) was found to be positively correlated to mean annual increment, but negatively to wood density and earlywood density. Similar correlation patterns were previously obtained between vulnerability to cavitation of mature wood and hydraulic conductivity to wood density and tree diameter (Rosner *et al.* 2008) and were thus discussed as tradeoffs between hydraulic and mechanical stress.

### Significant genetic markers

29 SNPs were identified to be responsible for 37 significant genetic associations for drought response, wood property traits and climate-growth correlations. These SNPs explain between 11% and 43% (Table 3) of the trait variation, which is relatively high as compared to other association studies. This pattern indicates strong divergent local selection of populations, which are connected by intermediate to high levels of gene flow as the low F_ST_ value at neutral nuclear genes indicates (Le Corre and Kremer 2012). In contrast, Beaulieu *et al.* (2011) analyzed 492 trees belonging to 165 families from 40 provenances in *Picea glauca* and significant markers accounted for 3–5% of the trait variation. Similar low values within broader genetic backgrounds were found for *Pinus taeda* L. (Brown *et al.* 2003; González-Martínez *et al.* 2007), *Pinus radiata* D. Don (Dillon *et al.* 2010), *Populus trichocarpa* Torr. & A. Gray (Porth *et al.* 2013) and *Eucalyptus globulus* (Thavamanikumar *et al.* 2014).

Associations across all provenances and across the SubsetQD resulted mainly in different SNPs associated with drought response and wood traits, but few SNPs were significant in both sets. Considering all provenances allowed for incorporating genetic variation within and across all provenances, while restricting associations to provenances with significant repeatabilities for drought removed a part of the phenotypic variation without a genetic basis, *i.e.*, the variation of provenances without significant repeatabilities. The remaining variation should mainly comprise genetic variation within provenances, which presumably did undergo natural selection to drought in their population history. Consequently, the amount of variation explained by the associated SNPs for SubsetQD was remarkably higher (R^2^ between 0.23 and 0.46) in comparison to the full dataset (R^2^ between 0.11 and 0.31). Both types of associations are important, in particular if SNPs were found to be associated with the same traits in both datasets.

GQ03709-C10.1.1133 (*Scen1*) and GQ03204-K15.1.405 (*Scen2*) were the only SNPs associated to binomial drought resistance traits with a strict classification of individuals in SubsetQD. GQ03709-C10.1.1133 is located downstream of a gene (MA_59794g0010) with an unknown function and GQ03204-K15.1.405 is located upstream of a Leucine Rich Repeat protein (MA_10434266g0010) as identified by its main Pfam domain (Table 4). LRRs are known to be involved in the formation of protein–protein interactions and are present in numerous proteins with diverse functions (Kobe and Kajava 2001). However, no similarity was found for MA_10434266g0010 by BLASTP. The absence of genotype “CC” in GQ03204-K15.1.405 could be caused by the low number of studied individuals as there is no distortion of Hardy-Weinberg equilibrium (Table S9). Although these SNPs are located in UTR, they might be important due to their possible function as regulatory elements on gene expression through differential transcription, siRNA targeting or mRNA stability (Webb *et al.* 2009). This assumption is supported by the gene expression profile of both genes, which are preferentially expressed in cambium and xylem expansion layers where new wood is being formed (Figure S8B).

We found higher numbers of associated SNPs for the drought events that had occurred in 2000 and 2003, while no associations were found for the drought event in 1993 (Table 3). At SNP WS-2.0-GQ02827.B7-M03.1-1062, genotypes AG were found to have better resilience within the SubsetQD as well as in the complete dataset. This marker is located in an untranslated region (UTR), but could have a function as regulatory element. A similar case was found for all provenances at SNP NODE-12228-length-276-cov-134.847824-162. SNPs found for recovery (*Rc00*) in all provenances were PGLM2-0082 and c89584_g2_i1_197. The first of these is located within a gene involved in transcription and the second one downstream (418 nt) of MA_90007g0010, an ARM repeat protein interacting with ABF2. This protein is involved in the abscisic acid (ABA) response and its involvement in drought response confirms the role of ABA as a plant hormone with an essential role in adaptation to various abiotic stresses including drought (Daszkowska-Golec 2016). The same SNP (c89584_g2_i1_197) was also associated to drought resistence in 2003. SNP ss538948434, which was found to be associated with recovery in the SubsetQD results in a non-synonymous substitution within the 3^rd^ exon of a pentatricopeptide repeat-containing protein (MA_100637g0010). This gene is highly expressed in new wood-forming tissue (cambium and xylem expansion layers; Figure S8) and is found to be associated with earlywood density (Lamara *et al.* 2016) in white spruce. Diverse functions have been attributed to this large family of modular RNA-binding proteins which mediate several aspects of gene expression primarily in organelles but also in the nucleus (Manna 2015).

Drought response indicators to the 2003 event were associated with ten SNPs in the complete dataset, but only with one SNP in the SubsetQD. The latter SNP ss538944271 is located in the intron of gene MA_161013g0010, which was identified as a putatively multicopper oxidase / L-ascorbate oxidase. This protein is known to oxidize ascorbate and was suggested to affect the plant system in response to stress deleteriously (Batth *et al.* 2017). Homozygous genotypes GG (of ss538944271) were found to show better recovery and this could be linked to a less effective or less expressed L-ascorbate oxidase. In Norway spruce, MA_161013g0010 belongs to a gene family (consisting of at least 24 members) and is preferentially expressed in cambium and xylem expansion layers (NorWood), where new wood is being formed (Figure S8B) and ascorbate might be needed under drought stress.

We also identified significant associations with climate-growth relationships that were inferred from traditional dendroclimatic analyses. The correlation between June temperature and annual increment is one of the most important climate-growth relationships in Norway spruce growing at lower elevations and at the warm margin of the species range (Schiessl *et al.* 2010). It demonstrates that high temperatures within the main growing period of spruce have a negative impact on annual increment. Hence, it is strikingly important to estimate the genetic variation in this relationship and to identify candidate genes which are involved in the response: All three SNPs with significant associations to *CorJunT* are located in exons and in all three markers, heterozygous genotypes show lower correlations between annual increment and June temperature compared to the homozygous genotypes. These heterozygous genotypes seem to be less sensitive to high summer temperatures and might be advantageous for growing in warm habitats under climate change. On the other hand, for all these three SNPs an homozygous genotype is absent (Table S9) but just the SNP GQ03204-O22.1.645 is out of Hardy-Weinberg equilibrium due to some reason we cannot ensure but putatively a deleterious effect of the homozygous absent allele. Among the three linked genes, GA2OX3 (Gibberellin 2-beta-dioxygenase 3) is the most interesting one as this protein is involved in the gibberellin biosynthesis, which is an important plant hormone for wood formation (Aloni 2013) and was found to be involved in the physiological response to drought (Zawaski and Busov 2014).

08Pg07115c is associated with two important wood traits in SubsetQD: *EW* and *RW*. This SNP marker is located 297 nucleotides (nt) downstream of a gene (MA_39636g0010) which is a transferase putatively involved in the lignin biosynthesis pathway (Table 4). Previous studies suggested that drought stress effects might be mitigated by plant secondary metabolism and lignification (Cabane *et al.* 2012). A BLASTP search of this gene indicated alignments to a hydroxycinnamoyl-CoA shikimate/quinate hydroxycinnamoyl transferase (HCT) within *Larix kaempferi*, *Pinus massoniana*, *Pinus pinaster* and *P. radiata* (Table S8). Compared to other tree species, Norway spruce has a large genome size (Nystedt *et al.* 2013) and this putatively HCT gene belongs to a family of 10 genes all putatively coding for HCT (Figure S9), probably with different levels of lignin biosynthesis activities and different substrate preferences (Kiselev *et al.* 2016). To test whether MA_39636g0010 coding protein still have the described function, more research would be necessary (Wagner *et al.* 2007). Nevertheless, there is strong evidence linking drought stress to modifications in lignification in different plants (Yoshimura *et al.* 2008), probably as a strategy to increase mechanical strength and/or water impermeability (Hu *et al.* 2009; Jubany-Mari *et a1*. 2009; Fromm 2013). Drought apparently can result in wall tightening and thickening, as observed in the tracheids of drought-stressed Norway spruce roots (Eldhuset *et al.* 2013). Tightening appears to be caused by a number of mechanisms, including lignification of wall polymers (Moore *et al.* 2008). An increased amount of lignin improves the mechanical strength of cell walls in a dry environment, and cell wall lignification helps to minimize water loss and cell dehydration (Cabane *et al.* 2012; Fang and Xiong 2015). So far, the MA_39636g0010 expression profile is not available at the NorWood platform, but HCT genes were found to be highly expressed during secondary growth and wood formation in conifers (Wagner *et al.* 2007). It should be noted that this SNP is out of Hardy-Weinberg equilibrium (Table S9) due to some reason we cannot ensure but putatively a deleterious effect of the homozygous genotype “CC”.

Wood density is likely to be influenced by lignin content and may be an important driver of the hydraulic properties in trees. It has been shown to affect conductivity and survival during drought in conifers such as Douglas-fir. (Martinez-Meier *et al.* 2008; Dalla-Salda *et al.* 2009). For SubsetQD, wood density (*RD*) was associated to a SNP (WS-2.0-GQ0036.TB-K03.1-397) directly located in the third exon of a putatively ERBB-3 binding protein 1, a gene that promotes organ growth by stimulating both cell proliferation and expansion (Horváth *et al.* 2006). Finally, for all provenances SNP 00930-O17-366 was associated to earlywood density (*ED*) resulting into a synonymous substitution being located in the unique exon of an unknown protein with no significant similarity found by BLASTP at present.

### Breeding Norway spruce in a changing climate

Norway spruce is the economic basis for sawnwood and pulp/paper production in many parts of Europe, particularly in Northern and Central European countries. Apart from Scandinavia, its high importance is not yet considered in breeding activities for more productive and climate resilient trees in Central Europe. The present study shows that the species’ Central and Southeastern European range harbors high genetic variation in drought response and in important wood characteristics, which could be further exploited in breeding schemes and genetic conservation programs. Given the high vulnerability of coniferous forests at the southern fringe of species ranges (*e.g.*, Schueler *et al.* 2014) and the low perception of such forests in nature conservation, the underlying basis of this variation is highly endangered. Thus, the given analysis is not only a step forward in the knowledge of the Norway spruce drought resistance and wood properties traits, but also in raising awareness to important adaptive variation that could get lost in the future. The restriction of our association study to provenances with significant genetic within-provenance variation is expected to avoid spurious false positive marker-phenotype associations. It allows us to provide a set of relevant genetic markers that could be useful (after validation in other populations) for breeding by marker assisted selection (MAS) and conservation genetics in a changing climate (Holliday *et al.* 2010) with future drought scenarios (Hamanishi and Campbell 2011). Moreover, we show that drought resistance as estimated from dendrochronological time series harbors sufficient degree of genetic determination and should be used as phenotypic trait within tree breeding and seed selection programs.

## Supplementary Material

Supplemental Material is available online at www.g3journal.org/lookup/suppl/doi:10.1534/g3.117.300524/-/DC1.
