# Supplementary material for: Drought Sensitivity of Norway Spruce at the Species’ Warmest Fringe: Quantitative and Molecular Analysis Reveals High Genetic Variation Among and Within Provenances
Source: G3 (Bethesda). 2018 Feb 9;8(4):1225–45. doi: 10.1534/g3.117.300524 (PMC5873913; doi:10.1534/g3.117.300524)
Supplement: Supplementary file 1 [file 1225FigureS1.pdf]

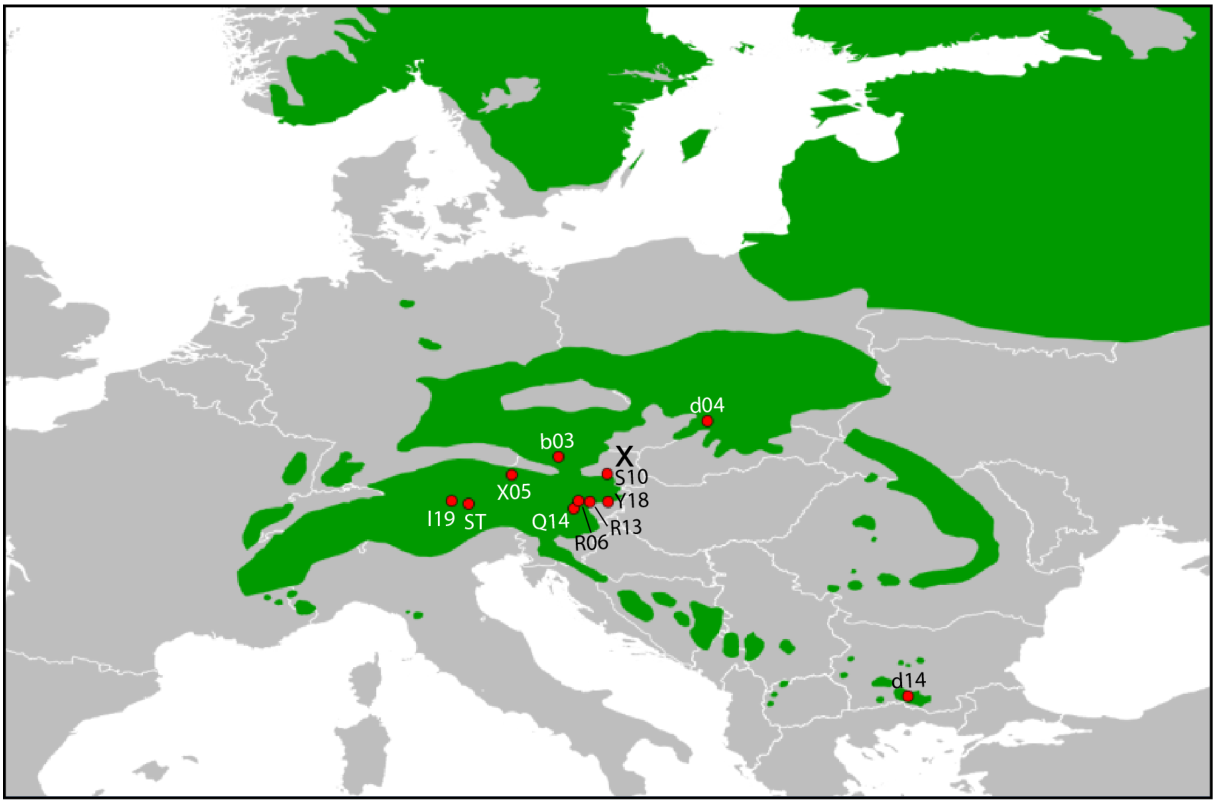

Figure S1. Geographic origin of the analysed provenances (red dots), location of the provenance trial (X) and natural distribution of Norway spruce (green area) according to EUFORGEN distribution maps.
