# Supplementary material for: Drought Sensitivity of Norway Spruce at the Species’ Warmest Fringe: Quantitative and Molecular Analysis Reveals High Genetic Variation Among and Within Provenances
Source: G3 (Bethesda). 2018 Feb 9;8(4):1225–45. doi: 10.1534/g3.117.300524 (PMC5873913; doi:10.1534/g3.117.300524)
Supplement: Supplementary file 2 [file 1225FigureS2.pdf]

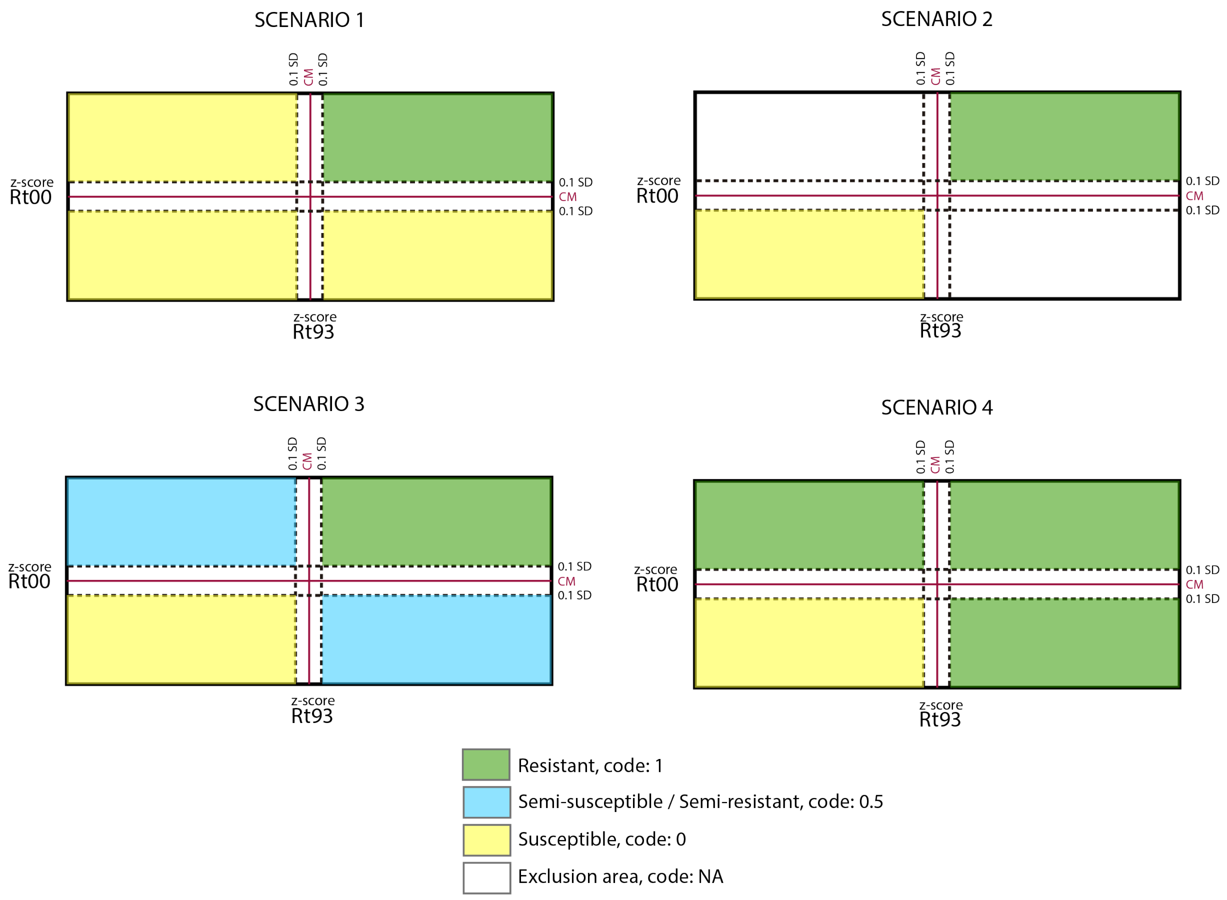

Figure S2. Scheme of different scenarios by combining ‘Resistance 1993’ and ‘Resistance 2000’ single-individual values transformed into z-scores and coded as follows for association analysis. Scenario 1: individuals with a z-score over the centered mean (CM) in both drought events (resistant, code: 1-green area) were distinguished from those with at least one z- score below the centered mean (susceptible, code: 0-yellow area). Scenario 2: individuals with z-score over the centered mean in both drought events (resistant, code: 1-green area) were distinguished from those with z-score below the centered mean in both drought events (susceptible, code: 0- yellow area). Individuals with z-scores over and a below the centered mean were excluded (NA) and not considered for the association analysis. Scenario 3: Similar to scenario 2, but in this case individuals with z-score over and a below the centered mean were defined as a third category called semi-susceptible/semi-resistant (code: 0.5-blue area). Scenario 4: individuals with at least one z-score over the centered mean (resistant, code: 1= green area) were distinguished from those with two z-score below the centered mean (susceptible, code: 0= yellow area). For all scenarios individuals with z-scores very close to the centered mean (CM) were excluded (NA) and not considered for the association analysis: cut-offs of 0.1 standard deviations around the centered mean were applied.
