# Supplementary material for: Drought Sensitivity of Norway Spruce at the Species’ Warmest Fringe: Quantitative and Molecular Analysis Reveals High Genetic Variation Among and Within Provenances
Source: G3 (Bethesda). 2018 Feb 9;8(4):1225–45. doi: 10.1534/g3.117.300524 (PMC5873913; doi:10.1534/g3.117.300524)
Supplement: Supplementary file 3 [file 1225FigureS3.pdf]

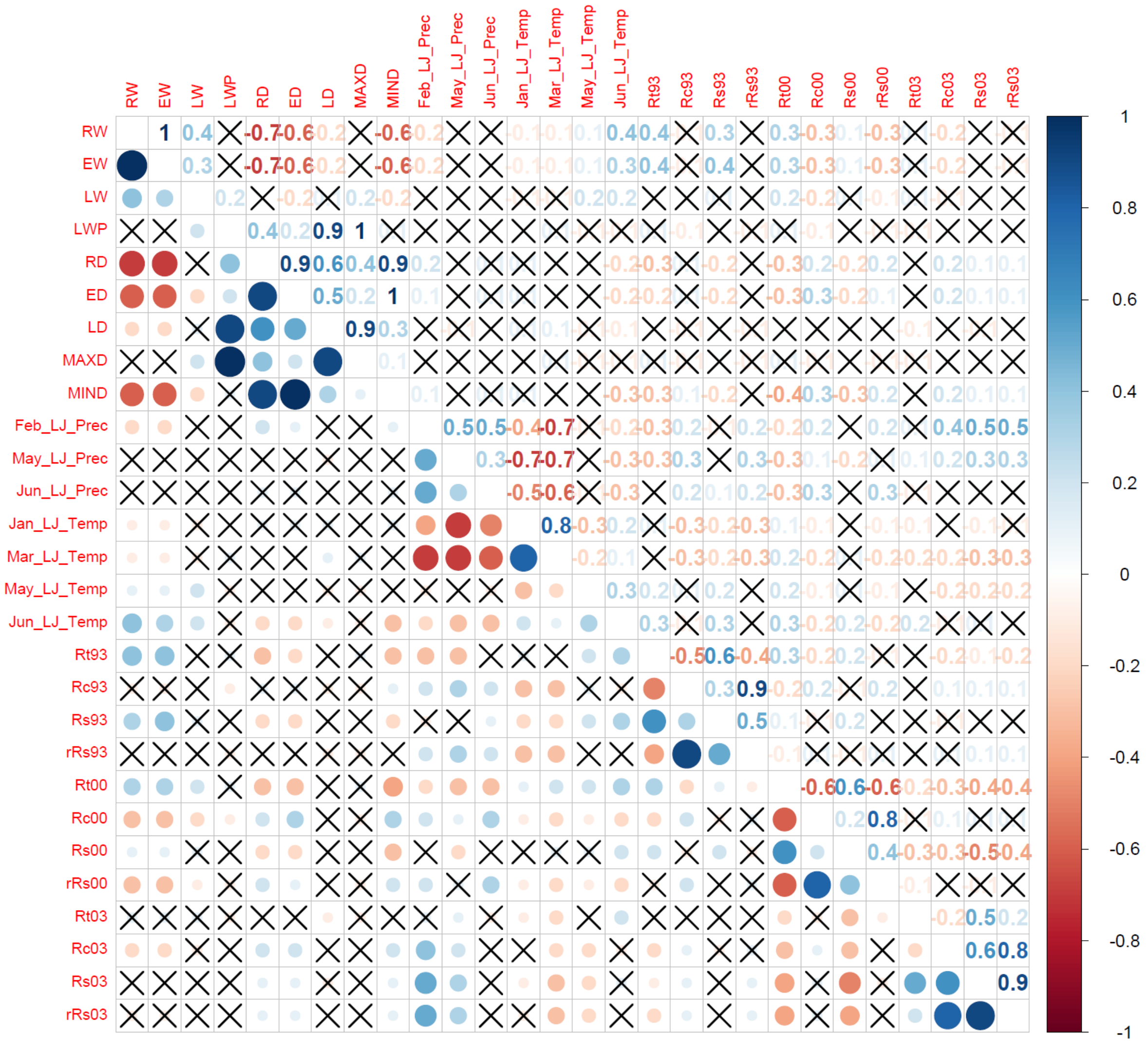

Figure S3. Overall correlation matrix for drought stress indicators, wood properties and climate-growth relationships. “X” in a cell means correlation is not significant at α < 0.05.
