# Supplementary material for: Drought Sensitivity of Norway Spruce at the Species’ Warmest Fringe: Quantitative and Molecular Analysis Reveals High Genetic Variation Among and Within Provenances
Source: G3 (Bethesda). 2018 Feb 9;8(4):1225–45. doi: 10.1534/g3.117.300524 (PMC5873913; doi:10.1534/g3.117.300524)
Supplement: Supplementary file 4 [file 1225FigureS4.pdf]

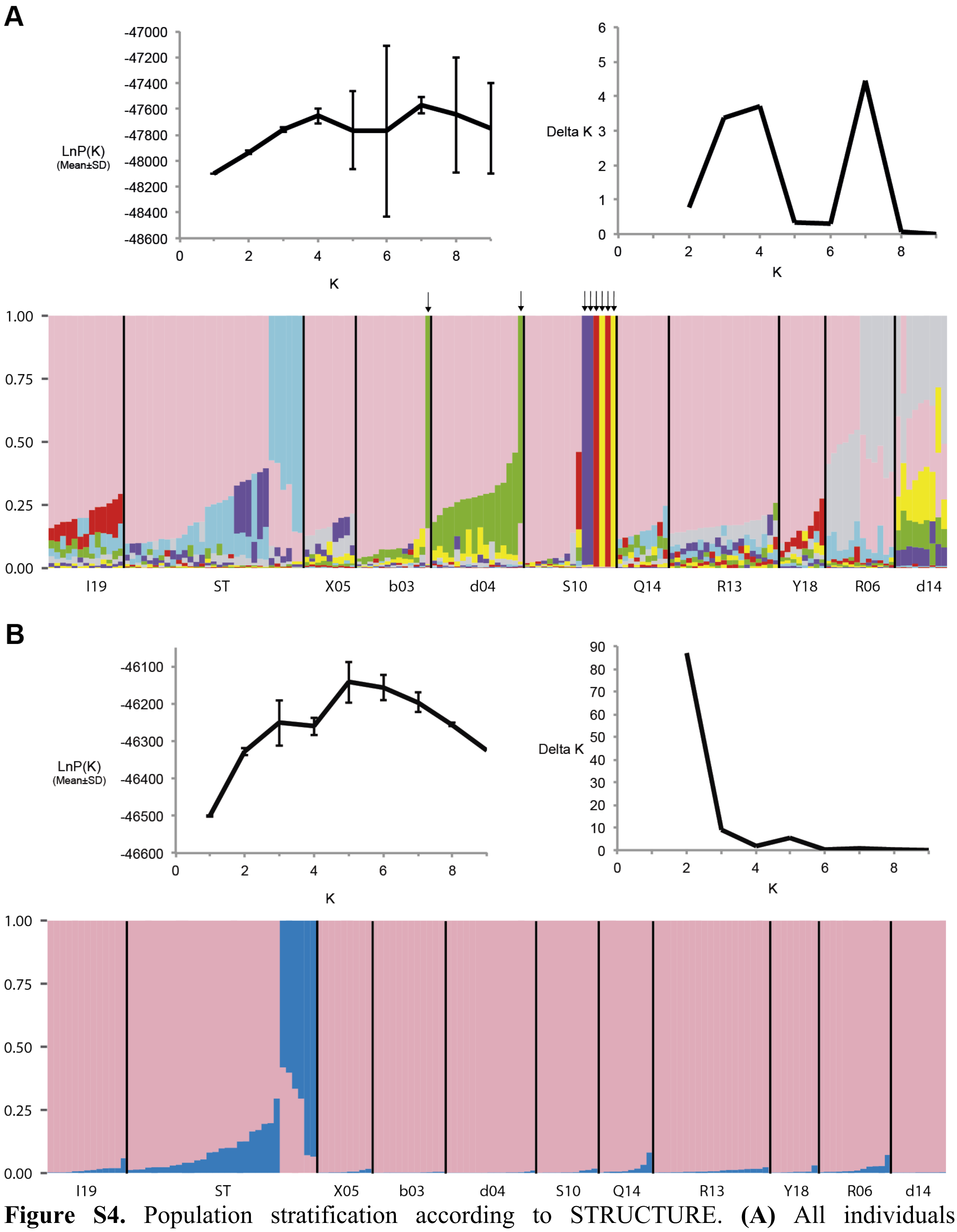

Figure S4. Population stratification according to STRUCTURE. **(A)** All individuals considered: 155 trees and 260 SNPs used, 8 trees (marked with black arrows) seems not to be related to the rest of the population: six individuals from location S10 as well one individual from d04 and one from b03. **(B)** 8 trees removed: 147 trees and 264 SNPs used. Shown are LnP (K), Delta K and bar plots, respectively. LnP (K) and Delta K graphs were obtained from Structure Harvester to estimate the most probable K number. In the bar plots each individual is represented by a single vertical box broken in colored segments according to the number of assumed population K=7 (155 trees) and K=2 (147 trees). Vertical box length is proportional to each cluster assignment probability (y-axis).
