# Supplementary figures and images for: Drought Sensitivity of Norway Spruce at the Species’ Warmest Fringe: Quantitative and Molecular Analysis Reveals High Genetic Variation Among and Within Provenances
Source: G3 (Bethesda). 2018 Feb 9;8(4):1225–45. doi: 10.1534/g3.117.300524 (PMC5873913; doi:10.1534/g3.117.300524)

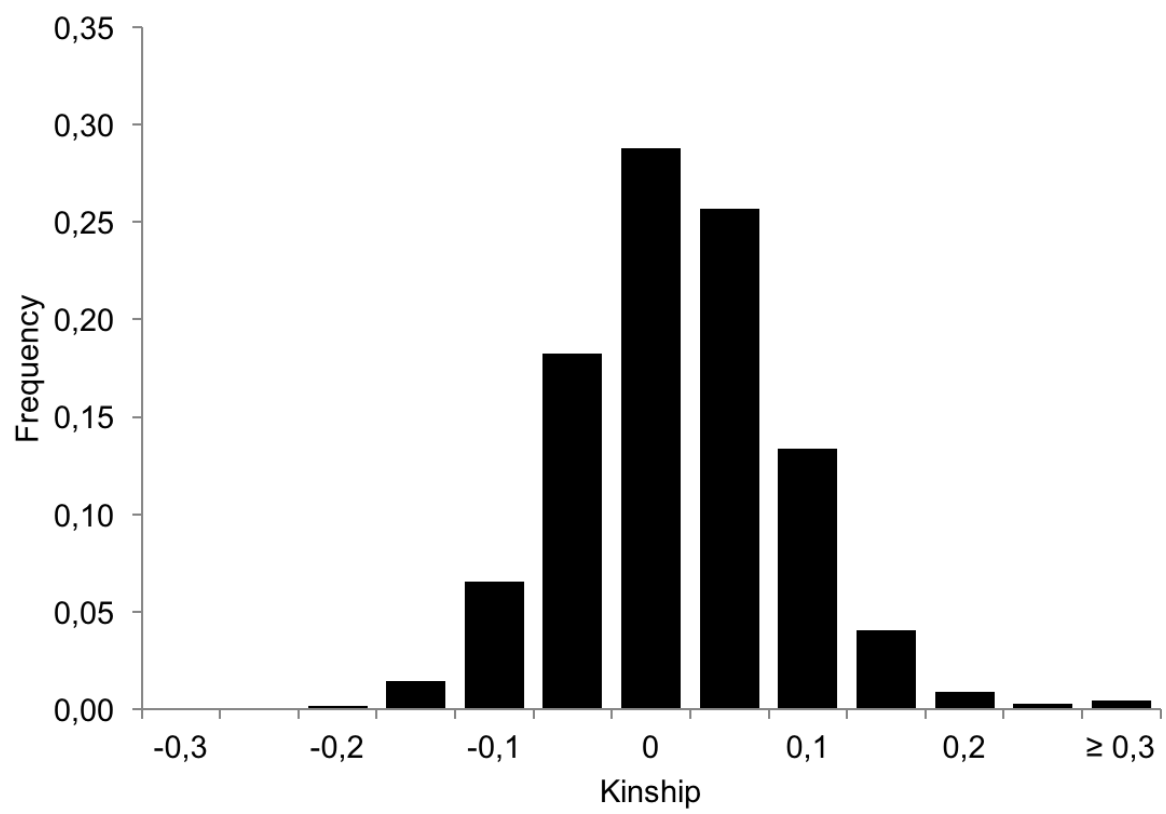

**Figure S5.** Frequency histogram of kinship matrix values.

Supplement: Supplementary file 5 [file 1225FigureS5.pdf]
