# Supplementary material for: Drought Sensitivity of Norway Spruce at the Species’ Warmest Fringe: Quantitative and Molecular Analysis Reveals High Genetic Variation Among and Within Provenances
Source: G3 (Bethesda). 2018 Feb 9;8(4):1225–45. doi: 10.1534/g3.117.300524 (PMC5873913; doi:10.1534/g3.117.300524)
Supplement: Supplementary file 6 [file 1225FigureS6.pdf]

A

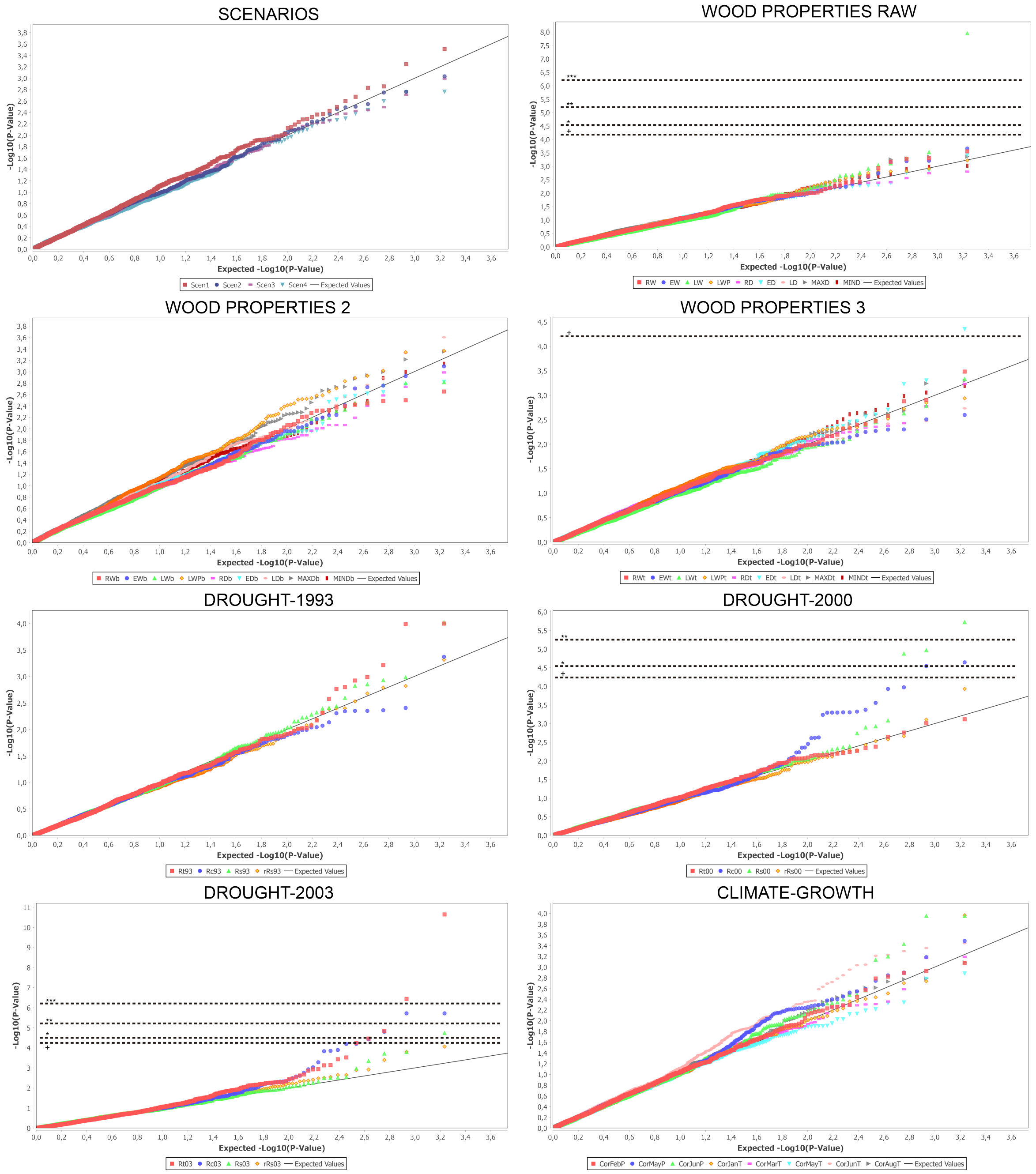
SCENARIOS
-Log10(P-Value)
Expected -Log10(P-Value)
Scen1 Scen2 Scen3 Scen4 Expected Values
WOOD PROPERTIES RAW
-Log10(P-Value)
Expected -Log10(P-Value)
RW EW LW LWP RD ED LD MAXD MIND Expected Values
WOOD PROPERTIES 2
-Log10(P-Value)
Expected -Log10(P-Value)
RWb EWb LWb LWPb RDb EDb LDb MAXDb MINDb Expected Values
WOOD PROPERTIES 3
-Log10(P-Value)
Expected -Log10(P-Value)
RWt EWt LWt LWPt RDt EDt LDt MAXDt MINDt Expected Values
DROUGHT-1993
-Log10(P-Value)
Expected -Log10(P-Value)
Rt93 Rc93 Rs93 rRs93 Expected Values
DROUGHT-2000
-Log10(P-Value)
Expected -Log10(P-Value)
Rt00 Rc00 Rs00 rRs00 Expected Values
DROUGHT-2003
-Log10(P-Value)
Expected -Log10(P-Value)
Rt03 Rc03 Rs03 rRs03 Expected Values
CLIMATE-GROWTH
-Log10(P-Value)
Expected -Log10(P-Value)
CorFebP CorMayP CorJunP CorJanT CorMarT CorMayT CorJunT CorAugT Expected Values

B

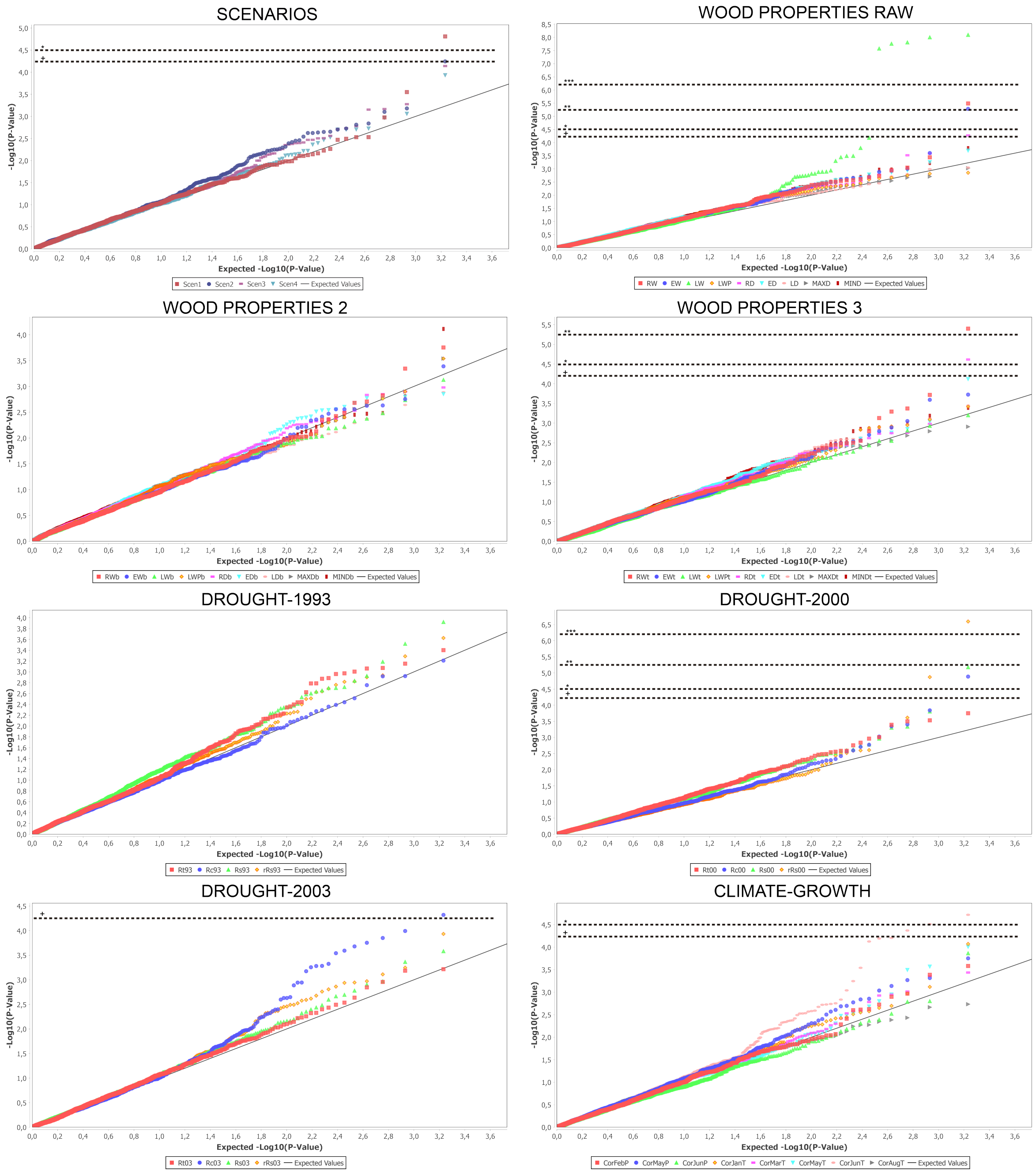

Figure S6. **(A)** Q-Q plots for All provenances MLM+Q+K. **(B)** Q-Q plots for SubsetQD MLM+Q+K. Horizontal dotted lines indicate the p-value threshold corrected with the standard Bonferroni procedure, resulting into the following corrected p-values for a given significance level α (α = ***0.001: $p < 5.83 \cdot 10^{-7}$; α = **0.01: $p < 5.83 \cdot 10^{-6}$; α = *0.05: $p < 2.92 \cdot 10^{-5}$; α = +0.1: $p < 5.83 \cdot 10^{-5}$) for all provenances and to (α = ***0.001: $p < 5.85 \cdot 10^{-7}$; α = **0.01: $p < 5.85 \cdot 10^{-6}$; α = *0.05: $p < 2$.
