# Supplementary material for: Drought Sensitivity of Norway Spruce at the Species’ Warmest Fringe: Quantitative and Molecular Analysis Reveals High Genetic Variation Among and Within Provenances
Source: G3 (Bethesda). 2018 Feb 9;8(4):1225–45. doi: 10.1534/g3.117.300524 (PMC5873913; doi:10.1534/g3.117.300524)
Supplement: Supplementary file 7 [file 1225FigureS7.pdf]

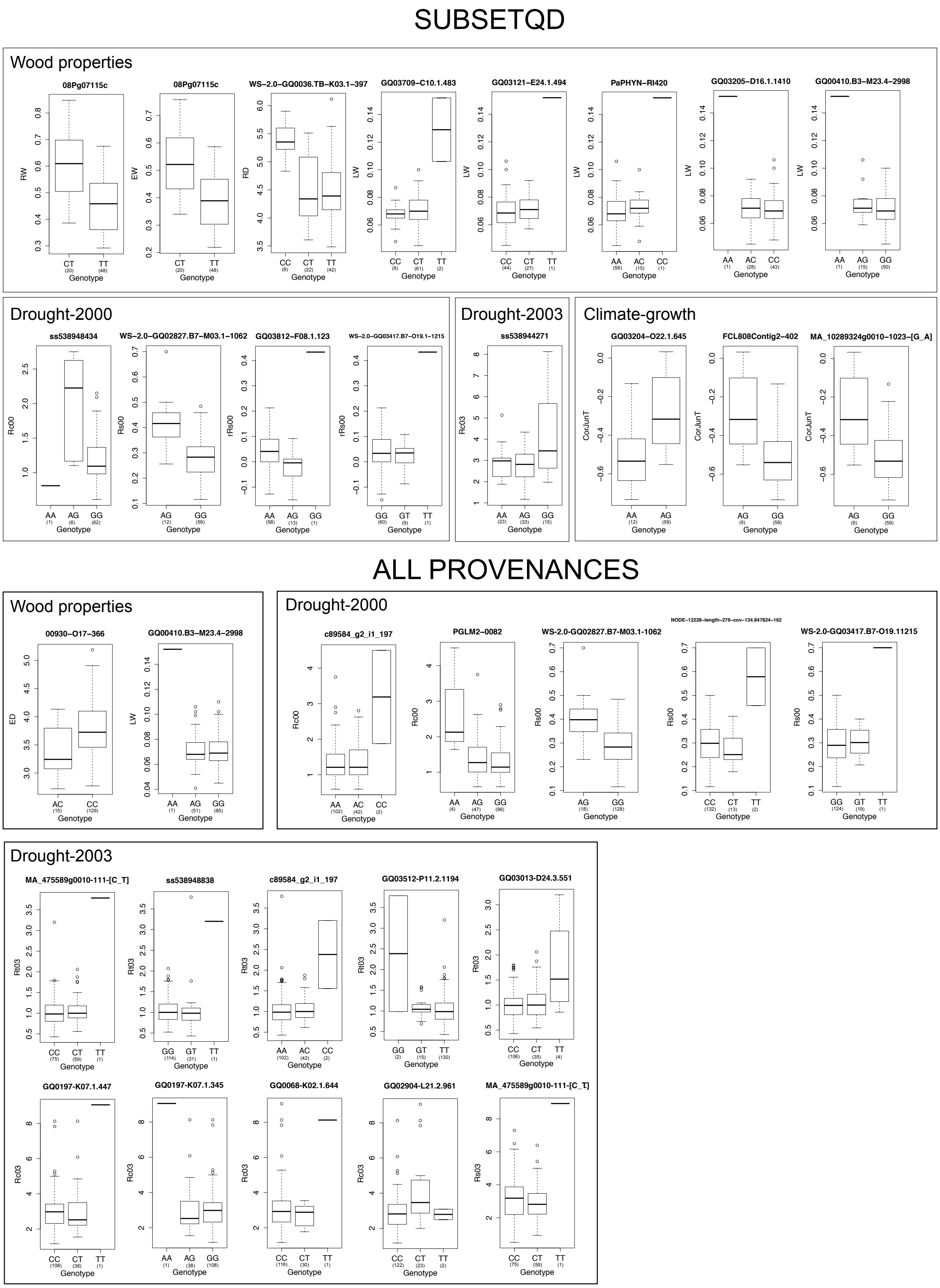

Figure S7. Box plot of wood, drought and climate-growth traits by genotypes in the associated SNP markers found for SubsetQD and all provenances. The boxes represent the median (black middle line) limited by the 25th (Q1) and 75th (Q3) percentiles. Numbers below each genotype indicates sample sizes.
