# Supplementary material for: Drought Sensitivity of Norway Spruce at the Species’ Warmest Fringe: Quantitative and Molecular Analysis Reveals High Genetic Variation Among and Within Provenances
Source: G3 (Bethesda). 2018 Feb 9;8(4):1225–45. doi: 10.1534/g3.117.300524 (PMC5873913; doi:10.1534/g3.117.300524)
Supplement: Supplementary file 8 [file 1225FigureS8.pdf]

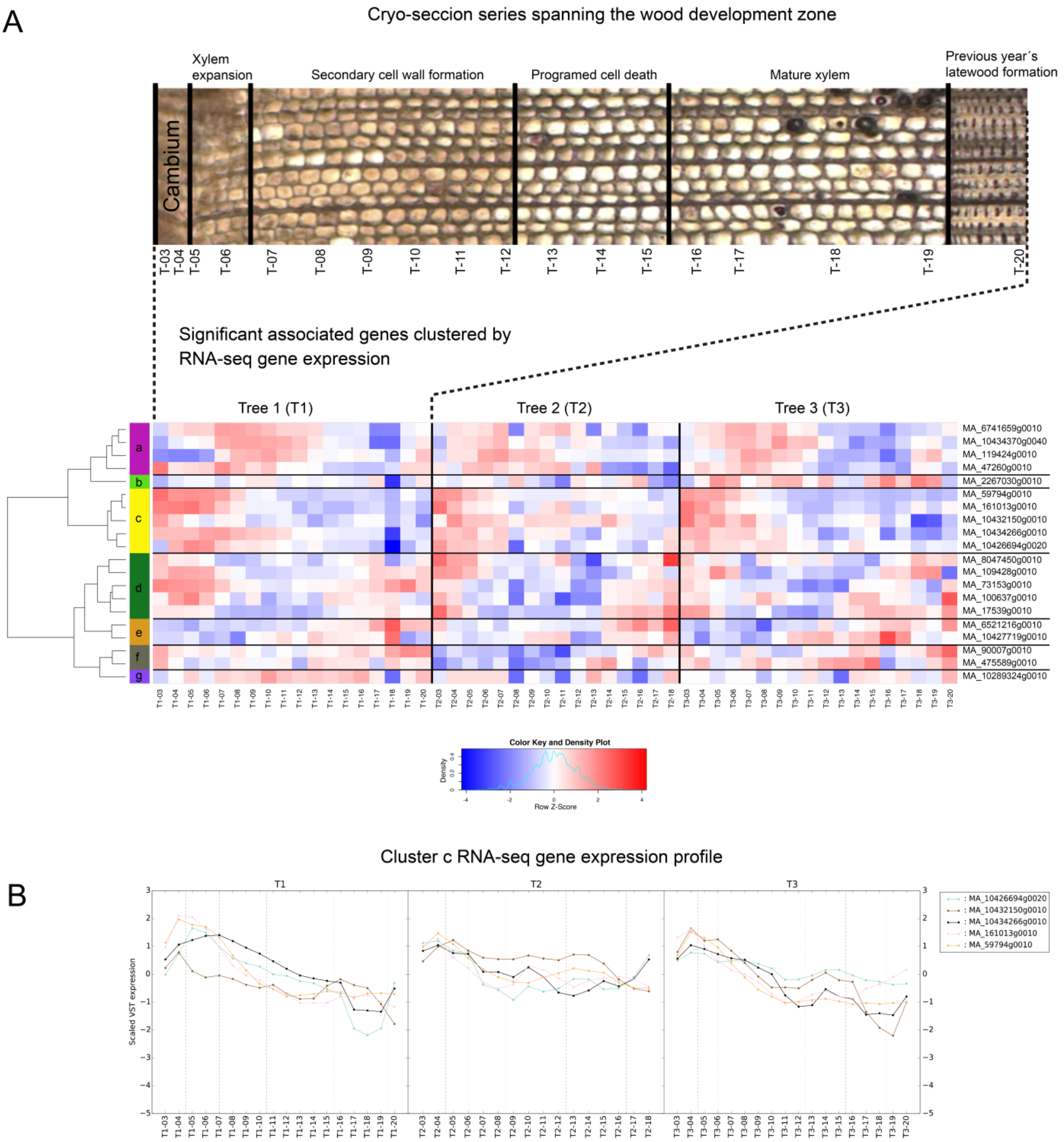

Figure S8. Clustering of associated gene expression profiles. **(A)** Hierarchical clustering of gene expression data from NorWood (http://norwood.congenie.org/) in cryo-section series of three trees. Seven gene expression clusters are indicated (a-g). Expression values are scaled per gene so that expression values above the gene average are represented by red, and below average by blue. **(B)** Detailed expression profiles of genes from cluster c: here genes are preferentially expressed in cambium and xylem expansion layers where new wood is being formed.
