# Supplementary material for: Drought Sensitivity of Norway Spruce at the Species’ Warmest Fringe: Quantitative and Molecular Analysis Reveals High Genetic Variation Among and Within Provenances
Source: G3 (Bethesda). 2018 Feb 9;8(4):1225–45. doi: 10.1534/g3.117.300524 (PMC5873913; doi:10.1534/g3.117.300524)
Supplement: Supplementary file 9 [file 1225FigureS9.pdf]

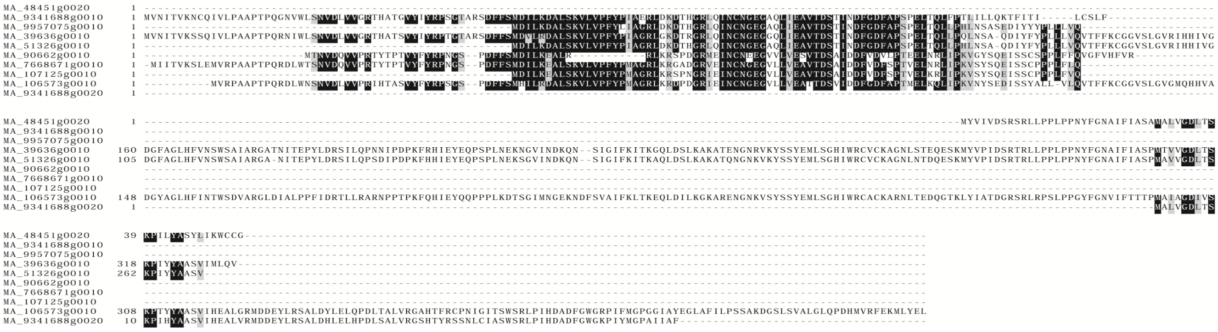

Figure S9. Multiple Sequence Alignment of 10 Norway spruce HCT putatively protein sequences (gene family 549; from www.congenie.org). Default settings on Clustal Omega at EMBL-EBI website (www.ebi.ac.uk/Tools/msa/clustalo) were used. Results were plot using BoxShade Server from ExPASy (www.ch.embnet.org/software/BOX_form.html).
